# Bridging the gap: the role of 3D cell cultures in mimicking tumor microenvironment for enhanced drug testing accuracy

**DOI:** 10.3389/fbioe.2025.1498141

**Published:** 2025-08-12

**Authors:** Yan Zhou, Feiyuan Yu, Min Guo, Yao Tang, Qian Xu

**Affiliations:** ^1^ Laboratory of Molecular Pathology, Department of Pathology, Shantou University Medical College, Shantou, China; ^2^ Department of Pathology, Huizhou First People’s Hospital, Huizhou, China; ^3^ Department of Cell Biology and Genetics, Shantou University Medical College, Shantou, China; ^4^ Stem Cell Clinical Research and Application Center, Zibo Central Hospital, Zibo, China; ^5^ Greenebaum Cancer Center, University of Maryland School of Medicine, Baltimore, MD, United States

**Keywords:** 3D cell culture, tumor microenvironment, organoids, drug sensitivity testing, drug screening

## Abstract

Cell culture is a crucial technology in life science research, particularly in cancer studies. The morphology and biological properties of tumor cells, along with the mechanisms of tumor development, are highly dependent on their culture conditions. Antitumor drug sensitivity testing is essential for cancer treatment, helping to identify effective therapies and reduce patient treatment burden. Currently, 2D cell culture remains the primary method for antitumor drug sensitivity testing due to its cost-effectiveness, ease of operation, and high-throughput screening capability. However, it does not accurately replicate the tumor microenvironment. Animal models are important tools for drug development, but they are not suitable for high-throughput screening. Recent advancements in 3D culture technologies have addressed this limitation. These technologies can better mimic the tumor microenvironment and can accurately reflect tumor biological behavior, gene expression, and signaling pathways. This paper summarizes the current *in vitro* and *in vivo* culture models, discusses emerging three-dimensional cell culture technologies, and highlights their ability to effectively simulate the tumor microenvironment and their significant potential in drug sensitivity testing.

## 1 Introduction

Drug sensitivity testing for antitumor drugs is a key method for assessing their efficacy and toxicity on tumor cells. Clinical guidelines for the use of antitumor drugs are generally based on the tumor type and stage of progression, often failing to account for individualized treatment. In addition, antitumor drugs have serious side effects, including bone marrow suppression and damage to the heart, liver, kidneys, and other organs. Accurate drug sensitivity testing can help improve drug efficacy, reduce patient suffering, and alleviate the financial and physical burden on patients. Therefore, a reliable tumor culture model is needed to evaluate the efficacy of candidate therapeutic drugs and develop personalized treatment plans.

Studies have shown that cell surface target expression and response to targeted drugs depend on the culture method (Kaur et al., 2021). Integrative analysis of drug transcriptomics has shown that gene expression profiles capture much of the variation in pharmacological profiles, suggesting the potential to develop predictive biomarkers based on gene expression to guide drug use (Bruun et al., 2020). Therefore, the choice of culture technique is crucial. The 2D culture model is easy to handle, highly standardized and reproducible, with straightforward data interpretation. These advantages make it suitable for high-throughput assays (Karlsson et al., 2012; Subia et al., 2021), which is why it remains the dominant model in antitumor drug research. However, traditional 2D culture lacks a three-dimensional growth environment and physiological conditions. For example, 2D cell culture cannot reproduce cell-cell communication or cell-matrix interactions (Fang and Eglen, 2017). Moreover, more aggressive subclones are selected during cell line establishment, and prolonged passaging leads to the accumulation of mutations (Deer et al., 2010). This means that the drug response of 2D-cultured cancer cells may not accurately reflect the behavior of tumors *in vivo*. Mouse models play an important role in drug screening and development. However, mouse models are expensive, time-consuming to establish, and not practical for high-throughput screening.

**FIGURE 1:**
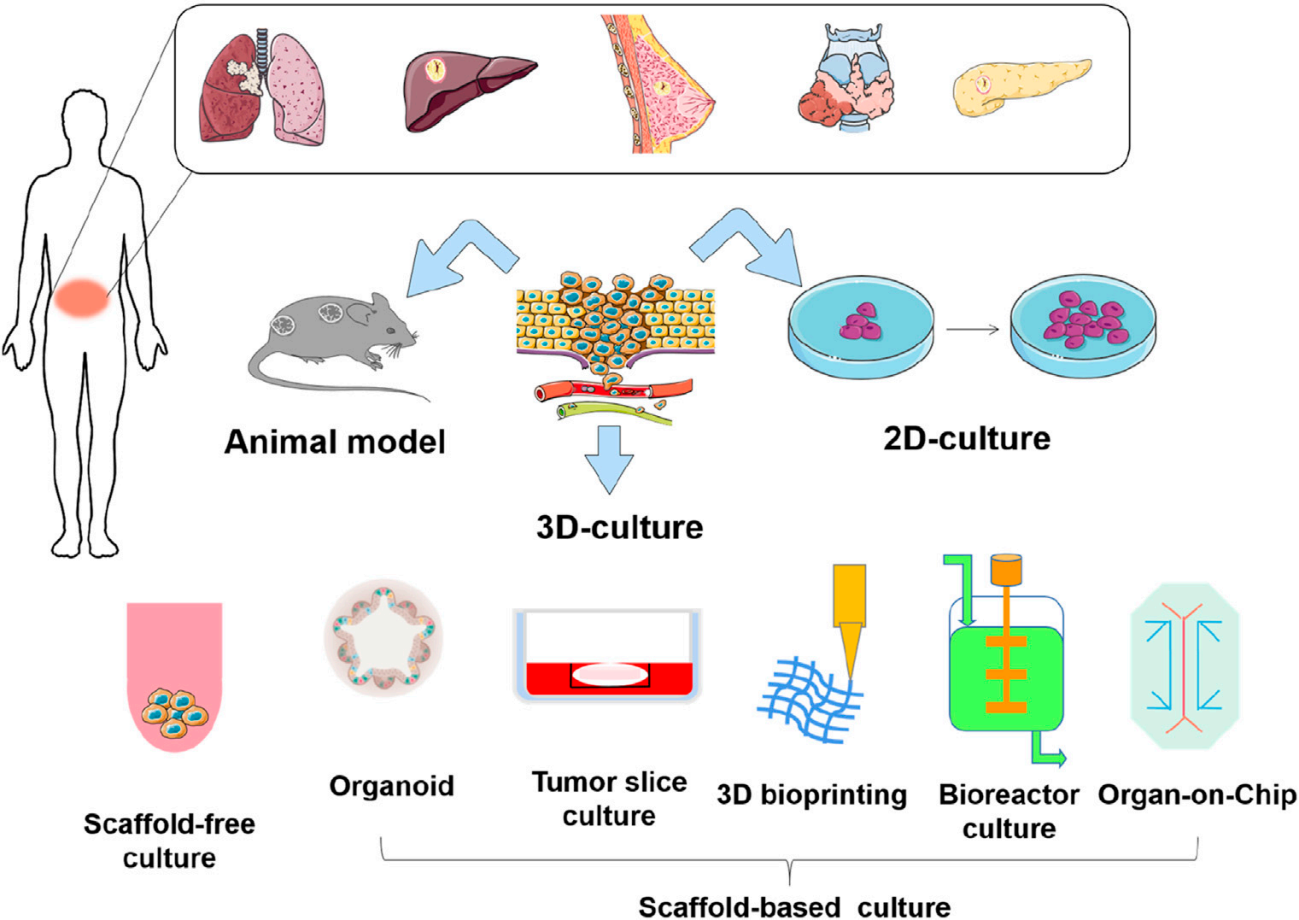
Overview of cell culture technology.

In recent years, advancements in bioengineering and biotechnology have led to the development of novel culture models, providing more options for evaluating the efficacy of antitumor drugs (Liao et al., 2019). Currently, 3D culture technologies include multicellular spheroids, organoids, organ-on-chip, and 3D bioprinting, each with its own advantages. Although these 3D culture techniques differ in their approaches, they can better mimic the morphology, functions, and microenvironment of cells *in vivo* and are more accurate in studying tumor progression and drug screening, compared with 2D culture (Qu et al., 2021; Ma et al., 2018; Boucherit et al., 2020). In this review, we systematically analyze the merits and limitations of current mainstream *in vitro* and *in vivo* culture paradigms (Figure 1). We highlight the application of these culture models in drug sensitivity testing, and incorporate the latest real-time monitoring technologies, such as the Seahorse XF Analyzer and D-OCT. Additionally, we discuss emerging applications of deep learning and artificial intelligence. We aim for this review can serve as a reference for researchers, provide valuable insights, and promote the development of *in vivo* and *in vitro* culture models for antitumor drug development and screening.

## 2 *In vitro* culture model

*In vitro* tumor culture is an important tool for screening anti-tumor drugs and evaluating treatment efficacy, encompassing monolayer cell culture, three-dimensional cell culture, organoid culture, organ-on-a-chip systems, bioreactor and so on. Cultured objects include primary tumor cells, tumor cell lines, fresh tumor tissue sections, tumor stem cells, *etc.* Cell culture-based drug sensitivity testing is a promising strategy. Currently available *in vitro* antitumor drug sensitivity testing includes both two-dimensional culture-based methods (CCK-8 assay, MTS assay, *etc.*) for assessing cell proliferation and survival, as well as apoptosis and cell cycle assays, along with collagen gel droplet-embedded culture drug sensitivity testing (CD-DST). These *in vitro* drug sensitivity tests may enable more precise and efficient prediction of tumor cell response to currently available anti-tumor drugs and molecularly targeted therapies under development, thereby informing their future clinical use (Miyazaki et al., 2016).

### 2.1 2D culture

The two-dimensional cell culture system is a flat-plate-supported monolayer cell culture system (Pampaloni et al., 2007). This system has been widely used since the early 20th century for research (Ferreira et al., 2018), particularly in studying cell heterogeneity through co-culture (Breslin and O’Driscoll, 2013). In 2D cell culture, cells are grown on a flat surface where they maintain direct contact with nutrients and growth factors in the culture medium. 2D cell culture techniques are popular among biologists and clinical researchers due to their simplicity and efficiency. In addition, the transwell culture system, a modified 2D culture system, has been developed as a co-culture system to simulate the *in vivo* environment (Hira et al., 2020; Noonan et al., 2019). However, these two-dimensional culture methods lack three-dimensional structures necessary for maintaining proper cell polarity and shape, and cannot recreate the complex tumor microenvironment. These limitations lead to altered gene expression and metabolism patterns - critical factors in antitumor drug sensitivity testing (Table 1).

**TABLE 1:** Differences between 2D culture and 3D culture in cellular characteristics.

Parameter	2D culture	3D culture
Cell morphology	Flat	Close to *in vivo* morphology
Cell growth	Rapid cell proliferation; Contact inhibition	Slow cell proliferation
Cell function	Functional simplification	Close to *in vivo* cell function
Cell communication	Limited cell-cell communication	Cell-cell communication, cell-matrix communication
Cell polarity and differentiation	Lack of polarity or even disappearance; incomplete differentiation	Maintain polarity; Normal differentiation

### 2.2 3D culture

In 1992, Petersen and Bissell used three-dimensional cell culture to simulate breast structures under cancerous and non-cancerous conditions (Petersen et al., 1992). Three-dimensional (3D) tumor culture models are now widely used to study tumorigenesis, *etc.* The major difference between 3D culture and 2D culture lies in the ability of 3D culture models to mimic the extracellular matrix (ECM) of native tissue. ECM is a dynamic protein network that maintains tissue homeostasis and cellular organization (Redmond et al., 2021). It is a scaffold composed of non-cellular fibronectin, various structural macromolecules and adhesion molecules that provide structural and biochemical support for cells and are involved in proliferation, adhesion, cell communication, and cell death (Henke et al., 2019; Wight et al., 1992). It is essential for many basic processes, such as cell differentiation and tissue repair (Schlie-Wolter et al., 2013). A variety of technologies have been derived from 3D culture in tumor research, including multicellular tumor spheroids, tumor-on-a-chip, and 3D bioprinting technologies. These methods generally take several weeks to establish functional models.

Three-dimensional cell models are established through two primary approaches: scaffold-free and scaffold-based culture methods. The scaffold-free culture approach cultivates cells in suspension, enabling them to self-assemble into the formation of multicellular spheroids (Flörkemeier et al., 2024). In this process, cell aggregation and growth occur solely through intrinsic cellular interactions, independent of external support structures. The scaffold-based culture method provides cells with a biocompatible carrier that is conducive to cell adhesion, proliferation, and migration. These scaffolds comprise either natural materials (e.g., collagen, Matrigel, and chitosan) or synthetic polymers (e.g., polycaprolactone) (Cortella et al., 2025; Risangud et al., 2024). Notably, currently used techniques like organoid culture and 3D bioprinting utilize scaffold-based systems, which constitute the primary focus of this review.

#### 2.2.1 Organoid

Organoids are established using the 3D cell culture system that enables stem cells to proliferate and differentiate into organ-like structures. These structures contain multiple cell types, have a spatial organization similar to their *in vivo* counterparts, and can recapitulate certain functions of the original organs. The foundation of the organoid culture system lies in the stem cells and the microenvironment. Based on the source of cells, organoids are mainly classified into normal tissue-derived organoids and tumor tissue-derived organoids. Normal tissue-derived organoids, cultured from pluripotent stem cells or adult stem cells, are currently used mainly for research on organ physiology. Tumor-derived organoids, established from tumor stem cells in culture and retain the heterogeneity of the original tumor. Organoids are widely used in cancer research, mainly for solid tumors. For non-solid tumors, such as blood tumors, the application of organoid technology still faces technical challenges (Xu et al., 2018).

Patient-derived tumor organoids (PDTOs) are established by culturing patient cancer cells in a 3D matrix. Extensive characterization demonstrates that PDTO models maintain greater similarity to the original tumor than 2D-cultured cells, while preserving genomic and transcriptomic stability (Beshiri et al., 2018), and bridging the gap between 2D cancer cell lines cultured *in vitro* and patient-derived tumor xenografts (PDTX) *in vivo* (Drost and Clevers, 2018; Sachs and Clevers, 2014). More importantly, they can be long-term expanded and cryopreserved, thus enabling the generation of biobanks of tumor organoids (van de Wetering et al., 2015). In cancer research, tumor-derived organoids retain the patient’s genetic alterations (Weeber et al., 2015). The 3D architecture of organoids more accurately recapitulates the histological and phenotypic characteristics of native tumors. Pasch et al. (2019) noted that patient-derived organoids can detect clonal heterogeneity with higher sensitivity than whole-tumor sequencing. For clinicians facing rare tumor cases where standard treatment guidelines are lacking, empirical drug testing often yields uncertain efficacy while potentially increasing treatment toxicity. In this context, patient-derived tumor organoids offer an efficient approach for high-throughput drug screening and personalized treatment optimization (Cao et al., 2022; Meier et al., 2022).

A key limitation in current organoid culture systems is the absence of a functional vascular network, which restricts oxygen and nutrient delivery to the organoid core (Nwokoye and Abilez, 2024). The vascular system plays a vital role in supplying nutrients and oxygen while also facilitating tumor metastasis. To better replicate these functions, researchers have developed vascularized tumor organoid models. These models include strategies such as coating organoids with endothelial cells or mesodermal progenitor cells to enable the spontaneous formation of capillary-like structures (Croft et al., 2019; Humpel, 2015). Another approach involves reprogramming mature endothelial cells into vasculogenic endothelial cells, which then integrate with decellularized tumor organoids to form functional vascular networks (Hutter-Schmid et al., 2015; Mielke et al., 2005). Additionally, vascularized organoids can be created using advanced 3D printing techniques to construct a fully integrated 3D vascular network (Sivakumar et al., 2019; Sönnichsen et al., 2018).

**FIGURE 2:**
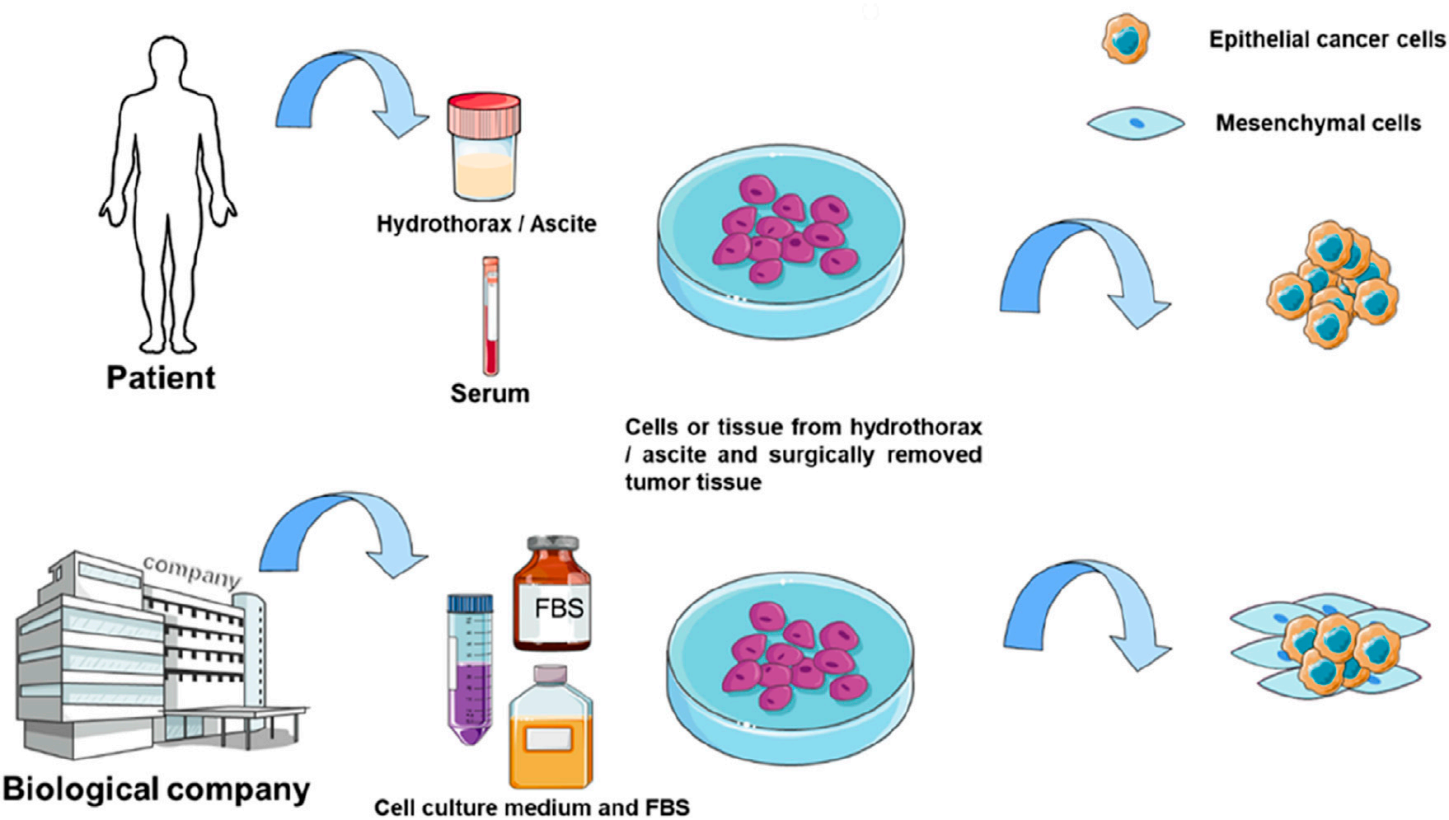
Comparison of 3D-ACM and 3D-FBS culture. The culture medium used for autologous culture is usually derived from patient serum or body fluids such as hydrothorax and ascite, while the traditional method used FBS for culture.

Autologous organoid culture represents an advanced method derived from organoid technology. Conventional organoid culture typically relies on commercial prepared media, mainly fetal bovine serum (FBS). Special growth factors are often added to culture medium to promote organoid formation (Wilson et al., 2014). This approach may ignore individual patient differences and the actual growth conditions of the tumor. Autologous culture uses the patient’s own serum or pleural effusion, ascites, to cultivate the patient’s own tumor cells. As a control, researchers cultured several patient-derived cancer samples under FBS-supplemented conditions and found that these conditions primarily supported the growth of mesenchymal stromal cells rather than epithelial cancer cells (Figure 2) (Tang et al., 2020a). This limitation affects subsequent studies based on such organoids, including drug sensitivity testing of antitumor drugs. These patient-specific fluids naturally contain the complete spectrum of nutrients, hormones, cytokines, and growth factors that sustain tumors in their native microenvironment - a biologically complex ecosystem impossible to replicate commercially. Tang et al. (2020a) applied 3D autologous culture (3D-ACM) to clinical specimens (including malignant effusions and surgical tumor tissue), demonstrating superior preservation of tissue architecture, immune profiles, and cytokine secretion compared to FBS-based cultures. Crucially, 3D-ACM maintained tumor biological properties more faithfully, as evidenced by more reliable chemosensitivity results, suggesting improved predictive accuracy for personalized treatment. Nevertheless, autologous culture presents challenges. First, the body fluids derived from different patients make the culture process difficult to standardize. Second, cancer patients are often frail, and their serum is limited, making it challenging to obtain sufficient quantities for autologous culture in drug sensitivity testing. However, autologous thoracoabdominal fluid is a better source of body fluids and is usually disposed of as medical waste without any physical or economic impact on the patient.

#### 2.2.2 Organotypic tissue slice culture

**FIGURE 3:**
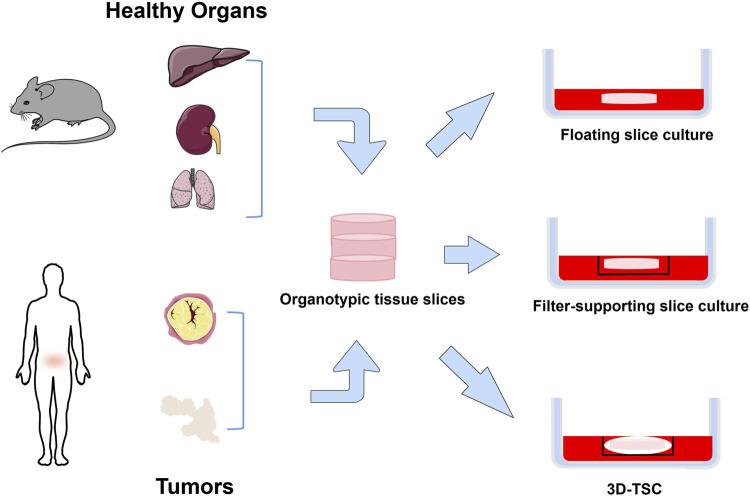
Organotypic tissue slice culture method. Normal or tumor tissue is obtained from experimental animals or humans, cut into thin slices, and placed in a dish for culture. Floating slice culture is placed directly in a culture dish. Filter-supporting slice culture is first placed on a filter and then placed in a culture dish. 3D-TSC is first wrapped slice with collagen, then placed in the culture inserts, and finally placed in a culture dish.

The organotypic tissue slice culture was first used in the 1970s for pharmacological evaluation (Willoughby et al., 1971). Surgically excised tissue is collected and placed in a cold medium, cut into cylinders or rectangles, and sectioned under sterile conditions within 6 h. Well-shaped sections were selected for culture (Cao et al., 2022) (Figure 3). Tumor slice culture (TSC) represents the closest model to the parental tumor because it retain the original tissue structure and cellular heterogeneity. Compared with organoids, the advantage of TSCs lies in the complex spatial organization and anatomical connectivity of intact tissue (Bahr, 1995; Croft et al., 2019). In addition, the tumor tissue culture model maintains vascular cells (Humpel, 2015; Hutter-Schmid et al., 2015). This is an advantage over primary cell line cultures or induced pluripotent stem cells (iPSC) cultures. Likewise, the specific genes and proteins expressed are maintained at levels comparable to those *in vivo* (Bahr, 1995; Mielke et al., 2005). Sections continue to grow for 10 days with a progressive increase in total viable cells, and key immune cell repertoire and gene expression levels of T and B lymphocytes can be fully preserved for at least 8 days (Cao et al., 2022). Three-dimensional tumor slice culture (3D-TSC) allows rapid and accurate replication of highly complex tumors, meanwhile, it use fluorescent-coupled antibodies and biopsy imaging to easily display multiple cell types (e.g., immune cells, endothelial cells, and cancer cells) and morphological structures (blood vessels and lymphatic vessels) in primary tumor sections, with the advantage of preserving cell repertoire and immune components, identifying tumor invasiveness, determining compound toxicity, rapid assessment of efficacy, and accurately predicting drug response (Sivakumar et al., 2019). More importantly, it can distinguish treatment responders from non-responders, providing a reliable tool for conducting drug sensitivity testing. This method enables the selection of optimal standard treatment plans for individualized therapy (Cao et al., 2022). This approach has been successfully implemented in colorectal cancer (Sönnichsen et al., 2018), breast cancer (Chakrabarty et al., 2022), head and neck squamous cancer (Gerlach et al., 2014), and human glioblastoma (Merz et al., 2013). However, the system is not a reproducible tool, and in addition, it is usually inefficient as it cannot test too many drug responses.

#### 2.2.3 3D bioprinting

Bioprinting is an advanced technology that utilizes 3D printing to create biological tissues and organs. Through precise 3D control, bioprinting technology can print cells, biomaterials and biofactors (i.e., bioinks) layer by layer to build tissues that are structurally and functionally similar to those in the body. Bioprinting provides an effective platform to model cancer angiogenesis and enable the construction of perfusable organoid models (Nwokoye and Abilez, 2024). The vascular network is a bridge that facilitates the exchange of gases, nutrients and waste products between the blood and surrounding cells (Monahan et al., 2013; Pittman, 2011), and is involved in the interactions between cells, extracellular matrix, and signaling molecules (Zhao et al., 2021; Naderi-Meshkin et al., 2023), which play an important role in the metastasis of tumors and the immune escape (Liu et al., 2024). At the same time, bioprinting is high-throughput and allows for efficient and standardized cell distribution (Kalla et al., 2024). Tumor heterogeneity is one of the mechanisms leading to drug resistance. The 3D bioprinting method can establish an *in vitro* model composed of different subtypes of tumor cells and non-tumor cells with controllable tumor microenvironment distribution (Moghimi et al., 2023). This is of great significance for the study of tumor heterogeneity and the exploration of chemotherapy resistance mechanisms.

#### 2.2.4 Bioreactor culture

A bioreactor is an engineered system designed for culturing cells, tissues, or microorganisms. It provides a more controlled environment to facilitate biological reactions and processes. Within bioreactors, drug sensitivity can be assessed more accurately by simulating *in vivo* conditions, including oxygen concentration, pH, temperature, and nutrient supply. It also enables continuous control and maintenance of culture conditions, improving the reproducibility of experiments. Ackermann et al. developed a xeno-free and chemically defined medium-scale bioreactor platform that enables continuous production of standardized human iPSC-derived hematopoietic-like organoids and macrophages (iPSC-Mac) (Ackermann et al., 2024). Moreover, the bioreactor can efficiently mimic the tumor microenvironment, making the biological behavior of tumor cells more plausible. De Luca et al. used a perfusion bioreactor to prepare scaffold morphologies with different pore sizes to reproduce Saos-2 cell behavior (De Luca et al., 2024). The bioreactor also provided continuous oxygenation and media perfusion to 3D cultured cells, promoting AEC-derived HCC to exhibit a stem cell phenotype (Campinoti et al., 2023). This bioreactor-driven ECM scaffold approach may enhance the functionality of pluripotent stem cells and support the development of more precise 3D cell culture systems (Campinoti et al., 2023).

#### 2.2.5 Organ-on-a-Chip

Micro-physiological systems, combined with tissue engineering, have facilitated the development of more physiologically relevant platforms, one of which is the Organ-on-a-Chip (OoC). The OoC platform is an advanced *in vitro* miniaturized precision-controlled bionic system designed to mimic the *in vivo* environment of cells and tissues and circulatory function (Bhatia and Ingber, 2014; Balijepalli and Sivaramakrishan, 2017; Peck et al., 2020). One of the most important advantages of this system is the ability to reproduce the key features of TME *in vitro*. Cellular interactions in TME often determine drug response and tumor fate. They are a major driver of tumor progression, and are potential therapeutic targets (Hanahan and Weinberg, 2011; Altorki et al., 2019; Grivennikov et al., 2010; Valkenburg et al., 2018). These organ-on-chip systems primarily consist of a cell culture chamber and channels for delivering the culture medium. By modulating the microchannels, they can simulate the structure and physiological state of solid tumor tissue, enabling drug evaluation and screening. The microchannels in the chip are also used to simulate capillaries *in vivo*, and the perfusion speed of the microchannels on the chip is adjusted to simulate the state of relatively insufficient vascular oxygenation in solid tumor tissues. After perfusion culture, the tumor tissue can be dissociated to analyze the metabolic state at different depths. Microfluidic chip culture can reflect the interaction between cells, cellular microenvironment, concentration gradient formed by various cytokines, etc., and with features such as high controllability, large-scale data generation, and reliable results (Polidoro et al., 2021). A key advantage of microfluidic chips is the ability to model the interplay between tumors, immune cells, and the vascular system, which plays a key role in tumor growth and immune escape (Schaaf et al., 2018). Recent research focus on the improvement of microfluidic devices and the optimization of drug combination screening schemes to meet clinical and industrial needs (Sun et al., 2018; Mulholland et al., 2018; Dorrigiv et al., 2023; Patra et al., 2016; Zhang et al., 2018). Microfluidic devices have achieved precise control of temperature, pH and other conditions to ensure the repeatability of experiments. These systems also integrate multiple functional modules, incorporate highly sensitive detection technologies, and utilize artificial intelligence to reduce operational complexity and improve screening efficiency.

3D cell culture methods have been widely adopted in cell biology, drug screening, and cancer research due to their ability to better mimic the growth environment of cells *in vivo*. However, reproducibility remains a critical challenge for the widespread application of 3D culture, particularly in preclinical research and drug development, where experimental consistency across studies is essential. The repeatability of 3D culture can be affected by tumor heterogeneity, culture materials, environmental conditions, and experimenters. Standardization of culture technology and the development of automated culture systems are conducive to improving repeatability. The 3D culture system combined with microfluidics technology can be used as a high-throughput screening tool in drug development. Utilizing automated operations, sensitive and rapid detection systems, and advanced data analysis platforms, these systems can test and analyze thousands of reactions simultaneously. This greatly enhances the scale and efficiency of drug screening, significantly reducing the time and costs associated with drug development (Yan et al., 2019).

## 3 *In vivo* culture model

New drugs must be tested in at least two animal species before they are allowed to be used in human clinical trials (Prior et al., 2018). Rodent models are widely used for preclinical studies because of their ease of handling, short growth cycles, low maintenance costs, and ease of gene editing (Saikawa et al., 1994). Currently, the major preclinical tumor models in mice include syngeneic mouse tumor models, genetically engineered mouse models (GEMMs), cell line-derived xenograft (CDX), patient-derived xenograft (PDX), and humanized mouse models.

Tumor patient-derived xenograft (PDX) models are established by implanting biopsy specimens, surgically resected tissue, malignant ascites-derived tumor cells, or circulating tumor cells (CTCs) into immunodeficient mice (Siolas and Hannon, 2013; Williams et al., 2015; Li et al., 2017). PDX models overcome many limitations of conventional cell line-derived xenografts (CDX), preserving the genetic and histological features, intratumoral heterogeneity, and tumor microenvironment (TME) of the original patient’s tumor (Hidalgo et al., 2014), traits that can persist even across successive generations in mice (Ding et al., 2010). This makes PDX models a useful tool for mechanistic studies and drug testing of cancer. However, recent studies highlight several limitations of PDX models. First, the engraftment success rate is lower than that of *in vitro* culture, the establishment time ranges from several weeks to several months, and the cost is high. Second, human tumor stromal cells and extracellular matrix are transplanted into immunodeficient mice, and ECM may gradually be replaced by murine components (Unger et al., 2014), compromising the TME and limiting their utility for cancer immunotherapy research. Finally, PDX relies on immunodeficient hosts, which restricts the evaluation of immunotherapies, and thus limits PDX’s applicability in the study of immunotherapy.

**FIGURE 4:**
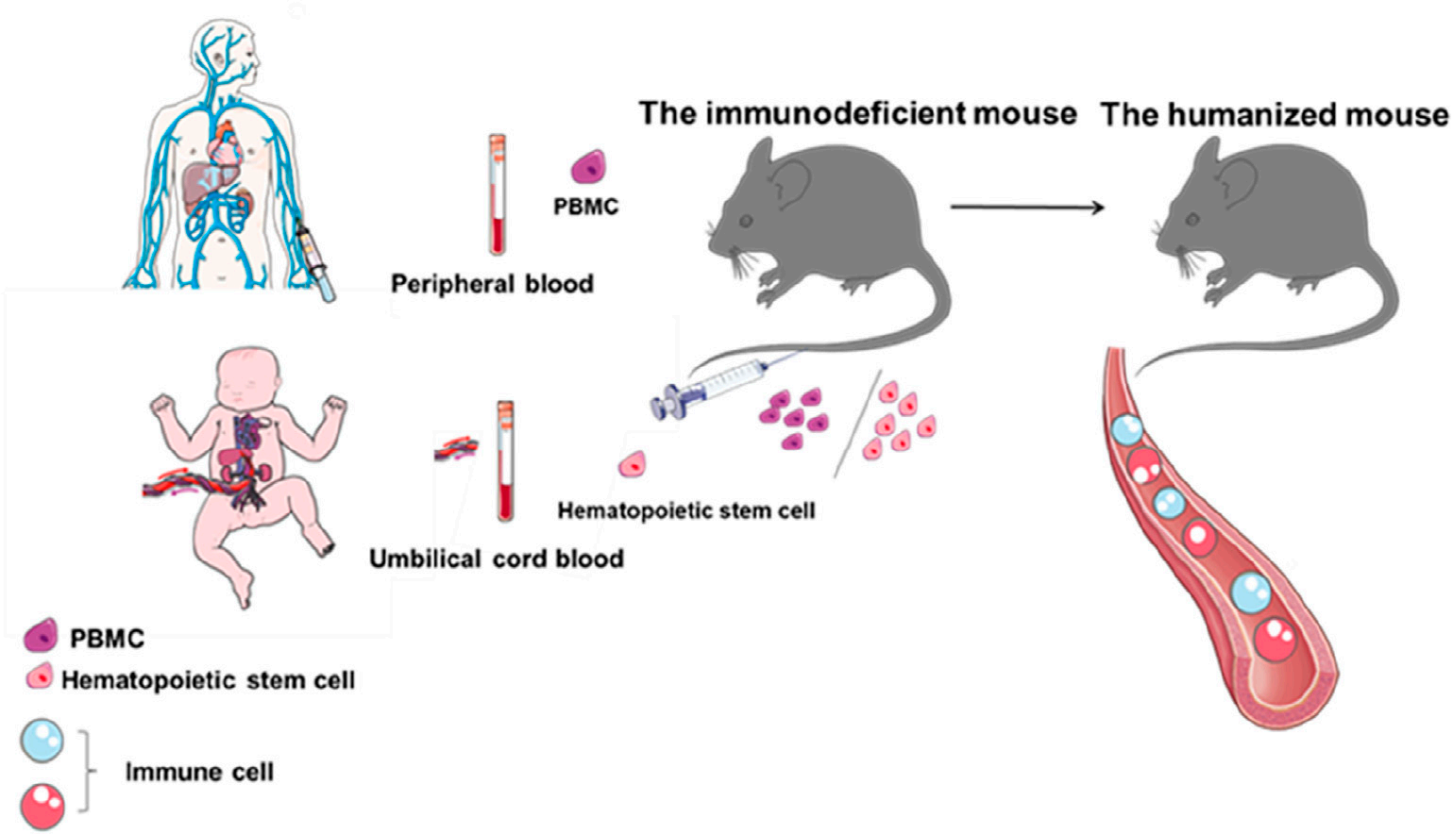
Flow chart of humanized mouse construction. Establishment of a mouse model with a human immune system by introducing peripheral blood mononuclear cells or hematopoietic stem cells from umbilical cord blood into immunodeficient mice.

Humanized hematopoietic stem cell (HSC) mouse models are generated by injecting human stem cells derived from umbilical cord blood or fetal tissue into immunodeficient mice with little or no functional immune system. These models reconstitute a functional human immune system, including T cells, B cells, and other immune cell populations, allowing researchers to directly study tumor biology and immune system function (Figure 4). However, humanized mice also have limitations. Establishing these models typically requires 8–12 weeks or longer, and the associated costs are high. In addition, graft-versus-host disease often occurs due to a major histocompatibility complex (MHC) mismatch between mouse hosts and human T lymphocytes (Franklin et al., 2022).

In general, animal models offer a stable physiological microenvironment for evaluating drug candidate efficacy in a physiologically relevant system. However, species-specific differences introduce uncontrollable variables, resulting in low success rates and poor reproducibility. In addition, the time and economic costs are high, ethical considerations are complex, and animal models are unsuitable for high-throughput screening, which limits their application in anticancer drug sensitivity testing (Table 2).

**TABLE 2:** A brief comparison of the strengths and limitations of *in vivo* and *in vitro* cell culture techniques.

Cell culture technology	Culture model	Advantages	Disadvantages	References
2D Culture model	Monolayer culture	High repeatability Low cost High-throughput; Easy to operate	Lack of tumor microenvironment; Monolayer cell culture	Duval et al. (2017)
3D Culture model	Organoid	Simulate the *in vivo* environment; Long-term maintain; Preserve tumor heterogeneity	Lack of immune system and vascular network	Clevers (2016)
	Autologous culture	Individualized	No standardized system	Tang et al. (2020a)
	Organotypic tissue slice culture	Retains some of the 3D anatomy intact; Fast modeling	Poor repeatability; Inefficiency	Willoughby et al. (1971)
	Organ-on-a-Chip	High-throughput; Microfluidic fine control; All-in-one study of multiple organs	High costs	Del Piccolo et al. (2021)
	3D bioprinting	Accuracy and controllability High-throughput; Efficient	Printing accuracy cannot be guaranteed Difficult to build complex tissues and organs	Nwokoye and Abilez (2024), Shukla et al. (2024)
	Bioreactor	Precise control by multiple sensors High-throughput; Automation	High costs Technically complex operation	Ackermann et al. (2024), Chen et al. (2024)
*In vivo* culture model	PDX	Provide *in vivo* environment; maintain tumor heterogeneity	Modeling takes a long time; High costs; Lacks immune system; Heterogeneous microenvironment	Yoshida (2020)
	The humanized mouse	Provide *in vivo* environment; maintain tumor heterogeneity Simulating the human immune environment	Modeling takes a long time; High costs Transplantation Rejection	Walsh et al. (2017)

## 4 Comparison of the advantages and disadvantages of different tumor models

### 4.1 Biological behavior of tumors

The biological behavior of tumor cells, such as proliferation, migration, invasion, and metastasis, is critical for validating drug effectiveness. Torisawa et al. (2005) compared MCF-7 cell proliferation in traditional culture flasks and *in silico* microarrays. The rate of cell proliferation in 3D culture was significantly lower than that in 2D culture, closely resembling *in vivo* conditions (Torisawa et al., 2004), though variability exists across techniques and cell types (Barbosa et al., 2021). When long-term drug sensitivity studies are required, especially in brain tissue (Giandomenico et al., 2021) or retinal tissue (Volkner et al., 2021), the 3D spheroid system is preferred due to its functional stability over weeks (Messner et al., 2013). Drug development and screening also face challenges because true efficacy and side effects often emerge only after prolonged observation. Recent advances in organoid technology enable long-term expansion, improving accuracy in assessing drug effectiveness and safety (Sato et al., 2011). Cell migration is another critical metric, especially for immunotherapy (Fitzgerald et al., 2020). Mark et al. demonstrated that while NK cells retain cytotoxicity in 2D, their efficacy drops 5.6-fold in 3D due to impaired migration (Mark et al., 2020). Huang et al. further highlighted how 3D-printed biomimetic microstructures (e.g., channel size, curvature) dictate tumor cell migration patterns, revealing that spatial constraints in 3D environments alter invasion strategies compared to 2D (Huang et al., 2014). Velez et al. expanded on this by showing that 3D collagen architectures induce conserved migratory and transcriptional programs in tumor cells, linked to vasculogenic mimicry—a process where aggressive cancer cells form fluid-conducting networks independent of blood vessels (Velez et al., 2017).

### 4.2 Cell state and tumor microenvironment

*In vivo* antitumor responses depend critically on tumor morphology/structure and the cellular components of the tumor microenvironment (TME), including various stromal and immune cells. The hepatocellular carcinoma (HCC) spheroid culture system (Liao et al., 2019) better recapitulates the nutrient/waste exchange gradients found *in vivo*, unlike the uniform access seen in artificial monolayer cultures (Fontoura et al., 2020). The intricate interactions between cells within the tumor microenvironment (TME) play a pivotal role in driving tumor progression and shaping the response to immunotherapeutic agents. The COC device developed by Chakrabarty et al. is particularly suitable for studying the immune response in tumors, where immune cells can be added in a controlled manner through the inflow of top or bottom channels to build the tumor microenvironment required for tumor cell growth. A key advantage of this system is its ability to quantitatively incorporate human immune components and measure responses in real time, enabling accurate and efficient simulation of the tumor growth environment (Chakrabarty et al., 2022). Autologous culture and air-liquid interface (ALI) culture techniques mix finely cut tumor tissues with stromal matrices, thereby preserving the original microenvironment—including immune and stromal cells—and maintaining the immune characteristics of the tumor (Gu et al., 2024). These culture models show great potential as predictive platforms for precision therapy to evaluate the effectiveness of tumor immunotherapy in cancer patients.

Liao et al. (2019) demonstrated that 3D cell culture significantly influences cell polarity, differentiation, signaling cascades, and gene-expression profiles compared to monolayer culture (Pinto et al., 2017). Torisawa et al. (2005) developed a three-dimensional culture system using an array of cell panels on a silicon chip, a culture technique that preserves the original growth characteristics of tumors while allowing control of cell polarity. These systems also establish oxygen gradients, which profoundly impact tumor biology. Hypoxia within tumors can compromise both conventional therapies and immunotherapy efficacy (Chouaib et al., 2017). More importantly, the oxygen gradient affects the sensitivity of tumor drugs by activating DNA damage repair proteins, altering cellular metabolism, and decreasing proliferation (Riffle and Hegde, 2017; Wang et al., 2018). Additionally, 3D cultures enhance the release of extracellular vesicles (e.g., exosomes) from cancer cells. These vesicles modulate diverse cell types within the TME, promoting tumor progression and influencing both local and systemic immune responses—thereby contributing to immunotherapy resistance (Szajnik et al., 2010; Eguchi et al., 2020; Xie et al., 2019; Hwang et al., 2019; Seo et al., 2018; Xavier et al., 2020; Dai et al., 2020). The gravitational microfluidic platform (GMP) (Wang et al., 2020) and the OoC system (Dsouza et al., 2022) leverage controlled fluid flow to enhance cell functionality, differentiation, and longevity.

### 4.3 Drug sensitivity of tumor cells

Compared to cancer cells in 2D culture systems, cancer cells in 3D culture systems exhibit altered morphology, structure, and signaling networks, which significantly influence drug responses. For example, 3D cell clusters/spheroids often develop multicellular resistance to antitumor drugs (Desoize and Jardillier, 2000). Liao et al. (2019) demonstrated this using patient-derived HCC cells, confirming greater drug resistance in 3D cultures. Liu et al. (2017) observed IFN resistance in B16 melanoma exclusively in 3D conditions. Muguruma et al. (2020) reported higher IC50 values for cisplatin, paclitaxel, and other drugs in 3D-cultured triple-negative breast cancer *versus* 2D cultures.

3D-cultured spheroids typically exhibit three distinct zones: a proliferative outer layer, a senescent middle zone, and a necrotic hypoxic core (Edmondson et al., 2014; Yamada and Cukierman, 2007). Tumor stem cells generated under hypoxic conditions overexpress ATP-binding cassette transporters and exhibit drug resistance (Weiswald et al., 2015; Bai et al., 2015). The acidic hypoxic core (mediated by lactate overproduction and carbonic anhydrase IX overexpression) (Amiri et al., 2016; Kazokaitė et al., 2018; Nunes et al., 2019) compromises cellular uptake of weak basic drugs (e.g., doxorubicin, vincristine) by impairing membrane permeability, enhancing chemoresistance (Nunes et al., 2019). More importantly, the oxygen gradient that develops in the tumor microenvironment shapes the tumor phenotype and influences drug sensitivity by activating DNA damage repair proteins, altering cellular metabolism, and reducing proliferation (Riffle and Hegde, 2017; Wang et al., 2018). For example, breast cancer cells cultured in 3D models exhibited greater resistance to doxorubicin and paclitaxel, which correlated with reduced PARP/caspase-3 cleavage and elevated hypoxia levels (Imamura et al., 2015). Baek et al. (2016a), Baek et al. (2016b) demonstrated that osteosarcoma spheroids develop drug-impermeable dense cores where ECM acts as a penetration barrier, elevating IC50 values *versus* 2D cultures - underscoring 3D models' superiority for drug sensitivity testing.

The behavior exhibited by cells in a 3D environment is closer to the *in vivo* conditions, allowing drug permeability and distribution to more accurately reflect their *in vivo* performance (Cardoso et al., 2023). In addition, 3D culture systems can more accurately evaluate the absorption, distribution, metabolism and excretion of drugs (Tchoryk et al., 2019). These parameters are critical for drug development, especially in the early stages to identify potential problems and thus reduce the risk of failure in late development. Particularly valuable for long-term exposure studies, 3D systems can reveal cumulative drug effects undetectable in short-term assays (Kaminska et al., 2021). Compared to *in vivo* models, using 3D culture models for drug sensitivity testing offers several advantages. They minimize animal use, reducing ethical concerns and costs (Lancaster and Knoblich, 2014), while enabling faster model establishment and high-throughput screening. This approach also effectively reduces the financial and time burden on patients.

## 5 3D culture in the application of anticancer drug sensitivity testing

It has been demonstrated that 3D-cultured cells exhibit drug responses more closely resembling *in vivo* behavior compared to monolayer cultures (Hagemann et al., 2017). The efficacy of anticancer drugs varies among individuals (Inoue et al., 2018). To address this, various drug sensitivity testing technologies based on 3D cell culture systems have been developed and applied (Saikawa et al., 1994; Brown and Markman, 1996; Kondo et al., 2000; Kubota et al., 1995; Yamaue et al., 2003; Takamura et al., 2002).

The collagen gel droplet-embedded culture drug sensitivity testing (CD-DST) was once a prominent method (Takamura et al., 2002; Koezuka et al., 1993; Kobayashi et al., 2001). Developed by Kobayashi in 1995 (Sakuma et al., 2020), this assay integrates three-dimensional cell culture, serum-free culture, and image colorimetric analysis technologies. CD-DST addressed numerous challenges associated with conventional drug sensitivity testing (Kobayashi, 2003). It was widely applied to various cancers including colorectal cancer, gastric cancer, lung cancer and breast cancer (Kobayashi et al., 1997). In recent years, with the rise of organoids, patient-derived organoids (PDOs) have been widely utilized for screening potential anticancer drugs due to their ability to maintain the heterogeneity of patients' tumors. Numerous studies have demonstrated that using PDOs to predict patients' drug sensitivity yields reliable results in multiple cancers, including colorectal cancer, gastric cancer, pancreatic cancer, bladder cancer, ovarian cancer (Vlachogiannis et al., 2018; Lee et al., 2018; Tiriac et al., 2018; Ganesh et al., 2019; Nan et al., 2020). Additionally, organoids are also employed to predict the toxic side effects of drugs on non-target tissues. For instance, liver and kidney organoids are used to evaluate the hepatotoxicity and nephrotoxicity of chemotherapy drugs (Andersson, 2017; Takasato et al., 2015).

The tumor slice culture (TSC) provides a unique tool for investigating tumor sensitivity to chemotherapeutic agents (Merz et al., 2013). Breast cancer tissue slices can remain viable for up to 7 days under standard culture conditions, enabling the assessment of tumor resistance or sensitivity to different chemotherapy regimens (Naipal et al., 2016). Chakrabarty et al. (2022) developed a microfluidic platform that evaluates patient treatment responses using tumor tissue slices through precise control of growth conditions. Nguyen et al. (2018) utilized an on-chip reconstituted immunocompetent tumor microenvironment to demonstrate that cancer-associated fibroblasts (CAFs) critically drive drug resistance and modulate immune evasion. The optimized chip-based organotypic culture (COC) platform sustains prolonged proliferative activity in breast and prostate cancer tissues without significant morphological or genetic alterations.

The application of 3D bioprinting technology in drug sensitivity testing offers new possibilities for precision medicine. This 3D bioprinting approach enables the construction of complex multicellular tissue models that can predict treatment response, maintain stem cell characteristics, and assess tumor invasiveness and drug resistance. Pharmaceutical giants such as Roche are utilizing 3D-printed “livers” to evaluate drug toxicity and detect liver injury caused by medications like trovafloxacin (Nguyen et al., 2016). Research has found that 3D-printed biomimetic microenvironments are conducive to the maturation and functional stability of liver cells induced from pluripotent stem cells. The selection of bioinks and the complex kidney structure pose significant challenges for *in vitro* reconstruction of kidneys and their microenvironments. Using kidney progenitor cells derived from pluripotent stem cells and kidney-derived extracellular matrix, 3D-printed “kidneys” are developed for high-throughput drug-induced nephrotoxicity assays (Lawlor et al., 2021). Tang et al. utilized a 3D bioprinting system to integrate glioma stem cells, astrocytes, neural stem cells, and optionally macrophages to create a dynamic multi-cellular biomimetic glioblastoma model. The findings demonstrate that the 3D bioprinting model more closely recapitulates the transcriptomic profiles of patient-derived glioblastoma tissues and is compatible with CRISPR-Cas9-based large-scale whole-genome screening methods (Tang et al., 2020b).

## 6 Discussion

Antitumor drug sensitivity testing is essential for screening suitable drugs for precision therapy. Selecting cell culture methods that accurately simulate the *in vivo* environment is the most critical step in drug sensitivity testing. Therefore, developing reliable tumor culture methods is crucial for anticancer drug development and application. Due to advantages such as simplicity, low cost, and high-throughput screening, 2D culture still remains widely used in antitumor drug development and screening. However, the clinical applicability of 2D primary cultures is constrained by methodological limitations, such as inconsistent drug response prediction and low culture success rates in certain tumor types (Kondo et al., 1966; Salmon et al., 1978). Moreover, monolayer cell culture cannot accurately mimic the *in vivo* tumor state (Breslin and O'Driscoll, 2013; Yamada and Cukierman, 2007; Weigelt et al., 2014; Lovitt et al., 2014; Rimann and Graf-Hausner, 2012; Hirschhaeuser et al., 2010), making it a suboptimal choice for drug sensitivity testing.

In addition to factors such as nutrient/oxygen gradients and drug diffusion capacity (Boucherit et al., 2020; Weiswald et al., 2015; Langhans, 2018), differences in drug sensitivity are often attributed to variations in microenvironment and gene expression profiles (Bruun et al., 2020; Farhat et al., 2021). Compared to 2D culture, 3D culture systems better preserve original tumor characteristics, simulate the *in vivo* tumor microenvironment, and provide a superior platform for drug screening. As a highly promising emerging technology, 3D culture techniques have led to the development of various advanced methods, including organoid culture, organ-on-a-chip, and 3D bioprinting. Currently, a variety of 3D culture models have been commercialized. Organoids have attracted the attention of pharmaceutical companies. Many companies are working to develop standardized organoid production processes to reduce costs and improve experimental consistency. Regulatory agencies such as the FDA have gradually recognized organ-on-a-chip technology as a supplementary method for drug development to promote commercialization (Low et al., 2021). Many companies and scientific research institutions are promoting the development of printing materials and equipment to advance the commercialization of 3D bioprinting products.

3D culture systems still face multiple challenges: traditional evaluation methods may not be suitable for 3D models (Bengtsson et al., 2021); reproducibility and standardization of culture protocols are limited; light penetration in 3D structures is poor; and cellular imaging within complex geometric architectures is challenging. Recent studies are actively addressing these limitations. Ooft and colleagues (Ooft et al., 2019) developed a growth rate -based classification tool that calculates drug effects per cell division, thereby eliminating confounding factors from cell proliferation rates. The optimized high-throughput confocal microscopy systems enable automated imaging and quantitative analysis of GFP reporter activity in spheroids (Hiemstra et al., 2019), enhancing image processing fidelity (Yang et al., 2020). In addition, metabolomics analyses can also be performed using gas chromatography-mass spectrometry (GC-MS) or liquid chromatography-mass spectrometry (LC-MS) (Klontzas et al., 2024; Pelosi et al., 2024). As a burgeoning tool in metabolic analysis, the Seahorse XF Analyzer is capable of providing real-time and dynamic monitoring of cellular energy metabolism (Ghiraldelli et al., 2025). For bioreactors, external physical and chemical sensors can be used for real-time monitoring. Han et al. developed a novel microfluidic platform for the flexible construction of 3D co-culture tumor models with spatio-temporal resolution, utilizing digital fabrication techniques such as rapid laser cutting of biocompatible polymethylmethacrylate (PMMA) and digital light processing(DLP)-based 3D bioprinting to enable precise drug sensitivity testing (Han et al., 2024). Electrical impedance tomography (EIT) enables real-time, non-destructive, label-free cell analysis, while label-free dynamic optical coherence tomography (D-OCT) can perform visualization and quantitative assessment (Wu et al., 2018; Abd El-Sadek et al., 2024; Abd El-Sadek et al., 2023). The application of these instruments in microfluidic chips helps to interpret the results of drug sensitivity testing. Chiang et al. introduced a deep learning model based on phase-contrast images, providing a cost-effective solution for continuous detection in microfluidic chips (Chiang et al., 2024). In addition, high-content phenotypic screens with multiple parameters can be used to assess cellular and subcellular responses to classify drugs and optimize 3D screening. This strategy provides integrated insights into drug mechanisms of action and system-level pathway dynamics in response to therapy, as evidenced by automated platforms for patient-derived disease models and real-time targeting of malignant plasticity in cancer (Boussaad et al., 2021; Esquer et al., 2021).

## 7 Conclusion

This review synthesizes recent advances in tumor cell culture methodologies, encompassing 2D, 3D, and *in vivo* models. We critically evaluate the strengths and limitations of each approach, with a focus on applications in drug sensitivity testing. Notably, these technologies are not mutually exclusive but can be synergistically integrated. The microfluidic platform, artificial intelligence, and machine learning technologies can be integrated with the 3D culture system to provide novel insights into drug sensitivity testing. Future directions should maximize the potential of existing technologies, refine established systems, and integrating them with cutting-edge approaches, such as multi-omics analyses. This approach will optimize patient-specific drug selection while simultaneously elucidating fundamental drug resistance mechanisms to inform novel clinical strategies.

## Glossary

3D cell culture: Three-Dimensional Cell Culture
CD–DST: Collagen Gel Droplet - embedded Culture Drug Sensitivity Testing
ECM: Extracellular matrix
PDTO: Patient-derived tumor organoid
PDTX: Patient-derived tumor xenografts
TSC: Tumor slice culture
ACM: Autologous culture method
TME: Tumor microenvironment
iPSC: induced Pluripotent Stem Cells
OoC: Organ-on-Chip
GEMMs: Genetically engineered mouse models
PDX: Patient-derived xenograft
CTCs: Circulating tumor cells
CDX: Cell line-derived xenograft
MHC: Major histocompatibility complex
HCC: Hepatocellular Carcinoma
PARP: Poly ADP-ribose polymerase
GC-MS: Gas chromatography-mass spectrometry
LC-MS: Liquid chromatography-mass spectrometry
EIT: Electrical impedance tomography
D-OCT: Dynamic optical coherence tomography

## References

[B1] Abd El-Sadek I. Morishita R. Mori T. Makita S. Mukherjee P. Matsusaka S. (2024). Label-free visualization and quantification of the drug-type-dependent response of tumor spheroids by dynamic optical coherence tomography. Sci. Rep. 14 (1), 3366. 10.1038/s41598-024-53171-4 38336794 PMC10858208

[B2] Abd El-Sadek I. Shen L. T. Mori T. Makita S. Mukherjee P. Lichtenegger A. (2023). Label-free drug response evaluation of human derived tumor spheroids using three-dimensional dynamic optical coherence tomography. Sci. Rep. 13 (1), 15377. 10.1038/s41598-023-41846-3 37717067 PMC10505213

[B3] Ackermann M. Saleh F. Abdin S. M. Rafiei Hashtchin A. Gensch I. Golgath J. (2024). Standardized generation of human iPSC-derived hematopoietic organoids and macrophages utilizing a benchtop bioreactor platform under fully defined conditions. Stem Cell Res. Ther. 15 (1), 171. 10.1186/s13287-024-03785-2 38886860 PMC11184717

[B4] Altorki N. K. Markowitz G. J. Gao D. Port J. L. Saxena A. Stiles B. (2019). The lung microenvironment: an important regulator of tumour growth and metastasis. Nat. Rev. Cancer 19 (1), 9–31. 10.1038/s41568-018-0081-9 30532012 PMC6749995

[B5] Amiri A. Le P. U. Moquin A. Machkalyan G. Petrecca K. Gillard J. W. (2016). “Inhibition of carbonic anhydrase IX in glioblastoma multiforme.” Eur. J. Pharm. Biopharm. 109, 81–92. 10.1016/j.ejpb.2016.09.018 27702686

[B6] Andersson T. B. (2017). Evolution of novel 3D culture systems for studies of human liver function and assessments of the hepatotoxicity of drugs and drug candidates. Basic and Clin. Pharmacol. Toxicol. 121 (4), 234–238. 10.1111/bcpt.12804 28470941

[B7] Baek N. Seo O. W. Kim M. Hulme J. (2016a). An SSA: Monmtoring the effects of doxorubicin on 3D-spheroid tumor cells in real-time. OncoTargets Ther. 9, 7207–7218. 10.2147/ott.s112566 PMC5125797 27920558

[B8] Baek N. Seo O. W. Lee J. Hulme J. An S. S. A. (2016b). Real-time monitoring of cisplatin cytotoxicity on three-dimensional spheroid tumor cells. Drug Des. Dev. Ther. 10, 2155–2165. 10.2147/dddt.s108004 PMC4938242 27445462

[B9] Bahr B. A. (1995). Long-term hippocampal slices: a model system for investigating synaptic mechanisms and pathologic processes. J. Neurosci. Res. 42 (3), 294–305. 10.1002/jnr.490420303 8583497

[B10] Bai C. Yang M. Fan Z. Li S. Gao T. Fang Z. (2015). Associations of chemo- and radio-resistant phenotypes with the gap junction, adhesion and extracellular matrix in a three-dimensional culture model of soft sarcoma. J. Exp. Clin. Cancer Res. 34 (1), 58. 10.1186/s13046-015-0175-0 26055407 PMC4467058

[B11] Balijepalli A. Sivaramakrishan V. (2017). Organs-on-chips: research and commercial perspectives. Drug Discov. Today 22 (2), 397–403. 10.1016/j.drudis.2016.11.009 27866008

[B12] Barbosa M. A. G. Xavier C. P. R. Pereira R. F. Petrikaitė V. Vasconcelos M. H. (2021). 3D cell culture models as recapitulators of the tumor microenvironment for the screening of anti-cancer drugs. Cancers 14 (1), 190. 10.3390/cancers14010190 35008353 PMC8749977

[B13] Bengtsson A. Andersson R. Rahm J. Ganganna K. Andersson B. Ansari D. (2021). Organoid technology for personalized pancreatic cancer therapy. Cell. Oncol. 44 (2), 251–260. 10.1007/s13402-021-00585-1 PMC7985124 33492660

[B14] Beshiri M. L. Tice C. M. Tran C. Nguyen H. M. Sowalsky A. G. Agarwal S. (2018). A PDX/organoid biobank of advanced prostate cancers captures genomic and phenotypic heterogeneity for disease modeling and therapeutic screening. Clin. Cancer Res. 24 (17), 4332–4345. 10.1158/1078-0432.ccr-18-0409 29748182 PMC6125202

[B15] Bhatia S. N. Ingber D. E. (2014). Microfluidic organs-on-chips. Nat. Biotechnol. 32 (8), 760–772. 10.1038/nbt.2989 25093883

[B16] Boucherit N. Gorvel L. Olive D. (2020). 3D tumor models and their use for the testing of immunotherapies. Front. Immunol. 11, 603640. 10.3389/fimmu.2020.603640 33362787 PMC7758240

[B17] Boussaad I. Cruciani G. Bolognin S. Antony P. Dording C. M. Kwon Y.-J. (2021). Integrated, automated maintenance, expansion and differentiation of 2D and 3D patient-derived cellular models for high throughput drug screening. Sci. Rep. 11 (1), 1439. 10.1038/s41598-021-81129-3 33446877 PMC7809482

[B18] Breslin S. O'Driscoll L. (2013). Three-dimensional cell culture: the missing link in drug discovery. Drug Discov. Today 18 (5-6), 240–249. 10.1016/j.drudis.2012.10.003 23073387

[B19] Brown E. Markman M. (1996). Tumor chemosensitivity and chemoresistance assays. Cancer 77 (6), 1020–1025. 10.1002/(sici)1097-0142(19960315)77:6<1020::aid-cncr3>3.0.co;2-l 8635118

[B20] Bruun J. Kryeziu K. Eide P. W. Moosavi S. H. Eilertsen I. A. Langerud J. (2020). Patient-derived organoids from multiple colorectal cancer liver metastases reveal moderate intra-patient pharmacotranscriptomic heterogeneity. Clin. Cancer Res. 26 (15), 4107–4119. 10.1158/1078-0432.ccr-19-3637 32299813

[B21] Campinoti S. Almeida B. Goudarzi N. Bencina S. Grundland Freile F. McQuitty C. (2023). Rat liver extracellular matrix and perfusion bioreactor culture promote human amnion epithelial cell differentiation towards hepatocyte-like cells. J. Tissue Eng. 14, 20417314231219813. 10.1177/20417314231219813 38143931 PMC10748678

[B22] Cao Y. Zhang X. Chen Q. Rao X. Qiu E. Wu G. (2022). Patient-derived organoid facilitating personalized medicine in gastrointestinal stromal tumor with liver metastasis: a case report. Front. Oncol. 12, 920762. 10.3389/fonc.2022.920762 35982969 PMC9378866

[B23] Cardoso B. D. Castanheira E. M. S. Lanceros-Mendez S. Cardoso V. F. (2023). Recent advances on cell culture platforms for in vitro drug screening and cell therapies: from conventional to microfluidic strategies. Adv. Healthc. Mater. 12 (18), e2202936. 10.1002/adhm.202202936 36898671 PMC11468737

[B24] Chakrabarty S. Quiros-Solano W. F. Kuijten M. M. P. Haspels B. Mallya S. Lo C. S. Y. (2022). A microfluidic cancer-on-chip platform predicts drug response using organotypic tumor slice culture. Cancer Res. 82 (3), 510–520. 10.1158/0008-5472.can-21-0799 34872965 PMC9397621

[B25] Chen W. Lu Y. Sun X. Leng J. Lin S. He X. (2024). A multifunctional CaCO3 bioreactor coated with coordination polymers enhances cancer immunotherapy. J. Control. Release 368, 780–796. 10.1016/j.jconrel.2024.03.026 38499091

[B26] Chiang C. C. Anne R. Chawla P. Shaw R. M. He S. Rock E. C. (2024). Deep learning unlocks label-free viability assessment of cancer spheroids in microfluidics. Lab a Chip 24 (12), 3169–3182. 10.1039/d4lc00197d PMC11165951 38804084

[B27] Chouaib S. Noman M. Z. Kosmatopoulos K. Curran M. A. (2017). Hypoxic stress: obstacles and opportunities for innovative immunotherapy of cancer. Oncogene 36 (4), 439–445. 10.1038/onc.2016.225 27345407 PMC5937267

[B28] Clevers H. (2016). Modeling development and disease with organoids. Cell 165 (7), 1586–1597. 10.1016/j.cell.2016.05.082 27315476

[B29] Cortella G. Lamparelli E. P. Ciardulli M. C. Lovecchio J. Giordano E. Maffulli N. (2025). ColMA-based bioprinted 3D scaffold allowed to study tenogenic events in human tendon stem cells. Bioeng. Transl. Med. 10 (1), e10723. 10.1002/btm2.10723 39801753 PMC11711214

[B30] Croft C. L. Futch H. S. Moore B. D. Golde T. E. (2019). Organotypic brain slice cultures to model neurodegenerative proteinopathies. Mol. Neurodegener. 14 (1), 45. 10.1186/s13024-019-0346-0 31791377 PMC6889333

[B31] Dai J. Su Y. Zhong S. Cong L. Liu B. Yang J. (2020). Exosomes: key players in cancer and potential therapeutic strategy. Signal Transduct. Target. Ther. 5 (1), 145. 10.1038/s41392-020-00261-0 32759948 PMC7406508

[B32] Deer E. L. Gonzalez-Hernandez J. Coursen J. D. Shea J. E. Ngatia J. Scaife C. L. (2010). Phenotype and genotype of pancreatic cancer cell lines. Pancreas 39 (4), 425–435. 10.1097/mpa.0b013e3181c15963 20418756 PMC2860631

[B33] Del Piccolo N. Shirure V. S. Bi Y. Goedegebuure S. P. Gholami S. Hughes C. C. W. (2021). Tumor-on-chip modeling of organ-specific cancer and metastasis. Adv. Drug Deliv. Rev. 175, 113798. 10.1016/j.addr.2021.05.008 34015419

[B34] De Luca A. Capuana E. Carbone C. Raimondi L. Carfì Pavia F. Brucato V. (2024). Three‐dimensional (3D) polylactic acid gradient scaffold to study the behavior of osteosarcoma cells under dynamic conditions. J. Biomed. Mater. Res. 112 (6), 841–851. 10.1002/jbm.a.37665 38185851

[B35] Desoize B. Jardillier J. (2000). Multicellular resistance: a paradigm for clinical resistance? Crit. Rev. Oncology/Hematology 36 (2-3), 193–207. 10.1016/s1040-8428(00)00086-x 11033306

[B36] Ding L. Ellis M. J. Li S. Larson D. E. Chen K. Wallis J. W. (2010). Genome remodelling in a basal-like breast cancer metastasis and xenograft. Nature 464 (7291), 999–1005. 10.1038/nature08989 20393555 PMC2872544

[B37] Dorrigiv D. Goyette P. A. St-Georges-Robillard A. Mes-Masson A. M. Gervais T. (2023). Pixelated microfluidics for drug screening on tumour spheroids and ex vivo microdissected tumour explants. Cancers 15 (4), 1060. 10.3390/cancers15041060 36831403 PMC9954565

[B38] Drost J. Clevers H. (2018). Organoids in cancer research. Nat. Rev. Cancer 18 (7), 407–418. 10.1038/s41568-018-0007-6 29692415

[B39] Dsouza V. L. Kuthethur R. Kabekkodu S. P. Chakrabarty S. (2022). Organ-on-Chip platforms to study tumor evolution and chemosensitivity. Biochimica Biophysica Acta Rev. Cancer 1877 (3), 188717. 10.1016/j.bbcan.2022.188717 35304293

[B40] Duval K. Grover H. Han L.-H. Mou Y. Pegoraro A. F. Fredberg J. (2017). Modeling physiological events in 2D vs. 3D cell culture. Physiol. (Bethesda, Md) 32 (4), 266–277. 10.1152/physiol.00036.2016 PMC5545611 28615311

[B41] Edmondson R. Broglie J. J. Adcock A. F. Yang L. (2014). Three-dimensional cell culture systems and their applications in drug discovery and cell-based biosensors. Assay Drug Dev. Technol. 12 (4), 207–218. 10.1089/adt.2014.573 24831787 PMC4026212

[B42] Eguchi T. Taha E. A. Calderwood S. K. Ono K. (2020). A novel model of cancer drug resistance: oncosomal release of cytotoxic and antibody-based drugs. Biology 9 (3), 47. 10.3390/biology9030047 32150875 PMC7150871

[B43] Esquer H. Zhou Q. Nemkov T. Abraham A. D. Rinaldetti S. Chen Y.-C. (2021). Isolating and targeting the real-time plasticity and malignant properties of epithelial-mesenchymal transition in cancer. Oncogene 40 (16), 2884–2897. 10.1038/s41388-021-01728-2 33742123 PMC8944243

[B44] Fang Y. Eglen R. M. (2017). Three-dimensional cell cultures in drug discovery and development. SLAS Discov. 22 (5), 456–472. 10.1177/1087057117696795 28520521 PMC5448717

[B45] Farhat J. Pandey I. AlWahsh M. (2021). Transcending toward advanced 3D-Cell culture modalities: a review about an emerging paradigm in translational oncology. Cells 10 (7), 1657. 10.3390/cells10071657 34359827 PMC8304089

[B46] Ferreira L. P. Gaspar V. M. Mano J. F. (2018). Design of spherically structured 3D *in vitro* tumor models -Advances and prospects. Acta Biomater. 75, 11–34. 10.1016/j.actbio.2018.05.034 29803007 PMC7617007

[B47] Fitzgerald A. A. Li E. Weiner L. M. (2020). 3D culture systems for exploring cancer immunology. Cancers 13 (1), 56. 10.3390/cancers13010056 33379189 PMC7795162

[B48] Flörkemeier I. Antons L. K. Weimer J. P. Hedemann N. Rogmans C. Krüger S. (2024). Multicellular ovarian cancer spheroids: novel 3D model to mimic tumour complexity. Sci. Rep. 14 (1), 23526. 10.1038/s41598-024-73680-6 39384844 PMC11464915

[B49] Fontoura J. C. Viezzer C. Dos Santos F. G. Ligabue R. A. Weinlich R. Puga R. D. (2020). Comparison of 2D and 3D cell culture models for cell growth, gene expression and drug resistance. Mater. Sci. Eng. C Mater. Biol. Appl. 107, 110264. 10.1016/j.msec.2019.110264 31761183

[B50] Franklin M. R. Platero S. Saini K. S. Curigliano G. Anderson S. (2022). Immuno-oncology trends: preclinical models, biomarkers, and clinical development. J. Immunother. Cancer 10 (1), e003231. 10.1136/jitc-2021-003231 35022192 PMC8756278

[B51] Ganesh K. Wu C. O’Rourke K. P. Szeglin B. C. Zheng Y. Sauve C. G. (2019). A rectal cancer organoid platform to study individual responses to chemoradiation. Nat. Med. 25 (10), 1607–1614. 10.1038/s41591-019-0584-2 31591597 PMC7385919

[B52] Gerlach M. M. Merz F. Wichmann G. Kubick C. Wittekind C. Lordick F. (2014). Slice cultures from head and neck squamous cell carcinoma: a novel test system for drug susceptibility and mechanisms of resistance. Br. J. Cancer 110 (2), 479–488. 10.1038/bjc.2013.700 24263061 PMC3899754

[B53] Ghiraldelli M. R. Machado I. F. Rolo A. P. Dorta D. J. Palmeira C. M. M. (2025). HepG2 spheroids cultured in alginate microcapsules as a model for exploring mitochondrial and glycolytic metabolism using the seahorse XFe24 analyzer. Toxicol. Mech. Methods, 1–9. 10.1080/15376516.2024.2447740 39757864

[B54] Giandomenico S. L. Sutcliffe M. Lancaster M. A. (2021). Generation and long-term culture of advanced cerebral organoids for studying later stages of neural development. Nat. Protoc. 16 (2), 579–602. 10.1038/s41596-020-00433-w 33328611 PMC7611064

[B55] Grivennikov S. I. Greten F. R. Karin M. (2010). Immunity, inflammation, and cancer. Cell 140 (6), 883–899. 10.1016/j.cell.2010.01.025 20303878 PMC2866629

[B56] Gu Z. R. Wu Q. Y. Shang B. Q. Zhang K. T. Zhang W. (2024). Organoid co-culture models of the tumor microenvironment promote precision medicine. Cancer Innov. 3 (1). 10.1002/cai2.101 PMC11212345 38948532

[B57] Hagemann J. Jacobi C. Hahn M. Schmid V. Welz C. Schwenk-Zieger S. (2017). Spheroid-based 3D cell cultures enable personalized therapy testing and drug discovery in head and neck cancer. Anticancer Res. 37 (5), 2201–2210. 10.21873/anticanres.11555 28476783

[B177] Han C. Zhang R. He X. Fang Y. Cen G. Wu W. (2024). A digital manufactured microfluidic platform for flexible construction of 3D co-culture tumor model with spatiotemporal resolution. Biofabrication 17 (1), 015008. 10.1088/1758-5090/ad9636 39577087

[B58] Hanahan D. Weinberg R. A. (2011). Hallmarks of cancer: the next generation. Cell 144 (5), 646–674. 10.1016/j.cell.2011.02.013 21376230

[B59] Henke E. Nandigama R. Ergün S. (2019). Extracellular matrix in the tumor microenvironment and its impact on cancer therapy. Front. Mol. Biosci. 6, 160. 10.3389/fmolb.2019.00160 32118030 PMC7025524

[B60] Hidalgo M. Amant F. Biankin A. V. Budinská E. Byrne A. T. Caldas C. (2014). Patient-derived xenograft models: an emerging platform for translational cancer research. Cancer Discov. 4 (9), 998–1013. 10.1158/2159-8290.cd-14-0001 25185190 PMC4167608

[B61] Hiemstra S. Ramaiahgari S. C. Wink S. Callegaro G. Coonen M. Meerman J. (2019). High-throughput confocal imaging of differentiated 3D liver-like spheroid cellular stress response reporters for identification of drug-induced liver injury liability. Arch. Toxicol. 93 (10), 2895–2911. 10.1007/s00204-019-02552-0 31552476

[B62] Hira V. V. Breznik B. Van Noorden C. J. Lah T. Molenaar R. J. (2020). 2D and 3D *in vitro* assays to quantify the invasive behavior of glioblastoma stem cells in response to SDF-1α. BioTechniques 69 (5), 339–346. 10.2144/btn-2020-0046 32867513

[B63] Hirschhaeuser F. Menne H. Dittfeld C. West J. Mueller-Klieser W. Kunz-Schughart L. A. (2010). Multicellular tumor spheroids: an underestimated tool is catching up again. J. Biotechnol. 148 (1), 3–15. 10.1016/j.jbiotec.2010.01.012 20097238

[B64] Huang T. Q. Qu X. Liu J. Chen S. (2014). 3D printing of biomimetic microstructures for cancer cell migration. Biomed. Microdevices 16 (1), 127–132. 10.1007/s10544-013-9812-6 24150602 PMC3945947

[B65] Humpel C. (2015). Organotypic brain slice cultures: a review. Neuroscience 305, 86–98. 10.1016/j.neuroscience.2015.07.086 26254240 PMC4699268

[B66] Hutter-Schmid B. Kniewallner K. M. Humpel C. (2015). Organotypic brain slice cultures as a model to study angiogenesis of brain vessels. Front. Cell Dev. Biol. 3, 52. 10.3389/fcell.2015.00052 26389117 PMC4557061

[B67] Hwang W.-L. Lan H.-Y. Cheng W.-C. Huang S.-C. Yang M.-H. (2019). Tumor stem-like cell-derived exosomal RNAs prime neutrophils for facilitating tumorigenesis of colon cancer. J. Hematol. Oncol. 12 (1), 10. 10.1186/s13045-019-0699-4 30683126 PMC6347849

[B68] Imamura Y. Mukohara T. Shimono Y. Funakoshi Y. Chayahara N. Toyoda M. (2015). Comparison of 2D- and 3D-culture models as drug-testing platforms in breast cancer. Oncol. Rep. 33 (4), 1837–1843. 10.3892/or.2015.3767 25634491

[B69] Inoue M. Maeda H. Takeuchi Y. Fukuhara K. Shintani Y. Funakoshi Y. (2018). Collagen gel droplet-embedded culture drug sensitivity test for adjuvant chemotherapy after complete resection of non-small-cell lung cancer. Surg. Today 48 (4), 380–387. 10.1007/s00595-017-1594-7 28993901

[B70] Kalla J. Pfneissl J. Mair T. Tran L. Egger G. (2024). A systematic review on the culture methods and applications of 3D tumoroids for cancer research and personalized medicine. Cell. Oncol. Dordr. 48, 1–26. 10.1007/s13402-024-00960-8 38806997 PMC11850459

[B71] Kaminska A. Wedzinska A. Kot M. Sarnowska A. (2021). Effect of long-term 3D spheroid culture on WJ-MSC. Cells 10 (4), 719. 10.3390/cells10040719 33804895 PMC8063822

[B72] Karlsson H. Fryknäs M. Larsson R. Nygren P. (2012). Loss of cancer drug activity in colon cancer HCT-116 cells during spheroid formation in a new 3-D spheroid cell culture system. Exp. Cell Res. 318 (13), 1577–1585. 10.1016/j.yexcr.2012.03.026 22487097

[B73] Kaur G. Doroshow J. H. Teicher B. A. (2021). Format (2D vs 3D) and media effect target expression and response of patient-derived and standard NSCLC lines to EGFR inhibitors. Cancer Treat. Res. Commun. 29, 100463. 10.1016/j.ctarc.2021.100463 34601320

[B74] Kazokaitė J. Niemans R. Dudutienė V. Becker H. M. Leitāns J. Zubrienė A. (2018). Novel fluorinated carbonic anhydrase IX inhibitors reduce hypoxia-induced acidification and clonogenic survival of cancer cells. Oncotarget 9 (42), 26800–26816. 10.18632/oncotarget.25508 29928486 PMC6003569

[B75] Klontzas M. E. Vernardis S. I. Batsali A. Papadogiannis F. Panoskaltsis N. Mantalaris A. (2024). Machine learning and metabolomics predict mesenchymal stem cell osteogenic differentiation in 2D and 3D cultures. J. Funct. Biomaterials 15 (12), 367. 10.3390/jfb15120367 PMC11680063 39728167

[B76] Kobayashi H. (2003). Development of a new in vitro chemosensitivity test using collagen gel droplet embedded culture and image analysis for clinical usefulness. Recent Results Cancer Res. 161, 48–61. 10.1007/978-3-642-19022-3_5 12528798

[B77] Kobayashi H. Higashiyama M. Minamigawa K. Tanisaka K. Takano T. Yokouchi H. (2001). Examination of *in vitro* chemosensitivity test using collagen gel droplet culture method with colorimetric endpoint quantification. Jpn. J. Cancer Res. Gann 92 (2), 203–210. 10.1111/j.1349-7006.2001.tb01083.x 11223550 PMC5926701

[B78] Kobayashi H. Tanisaka K. Doi O. Kodama K. Higashiyama M. Nakagawa H. (1997). An in vitro chemosensitivity test for solid human tumors using collagen gel droplet embedded cultures. Int. J. Oncol. 11 (3), 449–455. 10.3892/ijo.11.3.449 21528231

[B79] Koezuka M. Kondo N. Kobayashi H. Hara S. Yasutomi M. Nishida S. (1993). Drug sensitivity test for primary culture of human cancer-cells using collagen gel embedded culture and image-analysis. Int. J. Oncol. 2 (6), 953–959. 10.3892/ijo.2.6.953 21573652

[B80] Kondo T. Imamura T. Ichihashi H. (1966). In vitro test for sensitivity of tumor to carcinostatic agents. Gan 57 (2), 113–121. 4289605

[B81] Kondo T. Kubota T. Tanimura H. Yamaue H. Akiyama S. Maehara Y. (2000). Cumulative results of chemosensitivity tests for antitumor agents in Japan. Japan Research Society for Appropriate Cancer Chemotherapy. Anticancer Res. 20 (4), 2389–2392. 10953301

[B82] Kubota T. Sasano N. Abe O. Nakao I. Kawamura E. Saito T. (1995). Potential of the histoculture drug-response assay to contribute to cancer patient survival. Clin. Cancer Res. 1 (12), 1537–1543. 9815954

[B83] Lancaster M. A. Knoblich J. A. (2014). Organogenesis in a dish: modeling development and disease using organoid technologies. Science 345 (6194), 1247125. 10.1126/science.1247125 25035496

[B84] Langhans S. A. (2018). Three-dimensional *in vitro* cell culture models in drug discovery and drug repositioning. Front. Pharmacol. 9, 6. 10.3389/fphar.2018.00006 29410625 PMC5787088

[B85] Lawlor K. T. Vanslambrouck J. M. Higgins J. W. Chambon A. Bishard K. Arndt D. (2021). Cellular extrusion bioprinting improves kidney organoid reproducibility and conformation. Nat. Mater. 20 (2), 260–271. 10.1038/s41563-020-00853-9 33230326 PMC7855371

[B86] Lee S. H. Hu W. Matulay J. T. Silva M. V. Owczarek T. B. Kim K. (2018). Tumor evolution and drug response in patient-derived organoid models of bladder cancer. Cell 173 (2), 515–528.e17. 10.1016/j.cell.2018.03.017 29625057 PMC5890941

[B87] Li X. Zhu D. Li N. Yang H. Zhao Z. Li M. (2017). Characterization of ascites-derived tumor cells from an endometrial cancer patient. Cancer Sci. 108 (12), 2352–2357. 10.1111/cas.13407 28945304 PMC5715242

[B88] Liao W. Wang J. Xu J. You F. Pan M. Xu X. (2019). High-throughput three-dimensional spheroid tumor model using a novel stamp-like tool. J. Tissue Eng. 10, 2041731419889184. 10.1177/2041731419889184 31827757 PMC6886283

[B89] Liu Y. Liang X. Yin X. Lv J. Tang K. Ma J. (2017). Blockade of IDO-kynurenine-AhR metabolic circuitry abrogates IFN-γ-induced immunologic dormancy of tumor-repopulating cells. Nat. Commun. 8, 15207. 10.1038/ncomms15207 28488695 PMC5436221

[B90] Liu Y. Zhang X. Gu W. Su H. Wang X. Wang X. (2024). Unlocking the crucial role of cancer-associated fibroblasts in tumor metastasis: mechanisms and therapeutic prospects. J. Adv. Res. S2090-1232, 00220. 10.1016/j.jare.2024.05.031 PMC12126706 38825314

[B91] Lovitt C. J. Shelper T. B. Avery V. M. (2014). Advanced cell culture techniques for cancer drug discovery. Biology 3 (2), 345–367. 10.3390/biology3020345 24887773 PMC4085612

[B178] Low L. A. Mummery C. Berridge B. R. (2021). Organs-on-chips: into the next decade. Nat. Rev. Drug Discov. 20, 345–361. 10.1038/s41573-020-0079-3 32913334

[B92] Ma X. Liu J. Zhu W. Tang M. Lawrence N. Yu C. (2018). 3D bioprinting of functional tissue models for personalized drug screening and in vitro disease modeling. Adv. Drug Deliv. Rev. 132, 235–251. 10.1016/j.addr.2018.06.011 29935988 PMC6226327

[B93] Mark C. Czerwinski T. Roessner S. Mainka A. Hörsch F. Heublein L. (2020). Cryopreservation impairs 3-D migration and cytotoxicity of natural killer cells. Nat. Commun. 11 (1), 5224. 10.1038/s41467-020-19094-0 33067467 PMC7568558

[B94] Meier M. A. Nuciforo S. Coto-Llerena M. Gallon J. Matter M. S. Ercan C. (2022). Patient-derived tumor organoids for personalized medicine in a patient with rare hepatocellular carcinoma with neuroendocrine differentiation: a case report. Commun. Med. 2, 80. 10.1038/s43856-022-00150-3 35789568 PMC9249908

[B95] Merz F. Gaunitz F. Dehghani F. Renner C. Meixensberger J. Gutenberg A. (2013). Organotypic slice cultures of human glioblastoma reveal different susceptibilities to treatments. Neuro-Oncology 15 (6), 670–681. 10.1093/neuonc/not003 23576601 PMC3661091

[B96] Messner S. Agarkova I. Moritz W. Kelm JM. (2013). Multi-cell type human liver microtissues for hepatotoxicity testing. Arch. Toxicol. 87 (1), 209–213. 10.1007/s00204-012-0968-2 23143619 PMC3535351

[B97] Mielke J. G. Comas T. Woulfe J. Monette R. Chakravarthy B. Mealing G. A. R. (2005). Cytoskeletal, synaptic, and nuclear protein changes associated with rat interface organotypic hippocampal slice culture development. Brain Res. Dev. Brain Res. 160 (2), 275–286. 10.1016/j.devbrainres.2005.09.009 16271399

[B98] Miyazaki R. Anayama T. Hirohashi K. Okada H. Kume M. Orihashi K. (2016). In vitro drug sensitivity tests to predict molecular target drug responses in surgically resected lung cancer. PLoS One 11 (4), e0152665. 10.1371/journal.pone.0152665 27070423 PMC4829246

[B99] Moghimi N. Hosseini S. A. Dalan A. B. Mohammadrezaei D. Goldman A. Kohandel M. (2023). Controlled tumor heterogeneity in a co-culture system by 3D bio-printed tumor-on-chip model. Sci. Rep. 13 (1), 13648. 10.1038/s41598-023-40680-x 37607994 PMC10444838

[B100] Monahan-Earley R. Dvorak A. M. Aird W. C. (2013). Evolutionary origins of the blood vascular system and endothelium. J. Thrombosis Haemostasis 11 (Suppl. 1), 46–66. 10.1111/jth.12253 PMC5378490 23809110

[B101] Muguruma M. Teraoka S. Miyahara K. Ueda A. Asaoka M. Okazaki M. (2020). Differences in drug sensitivity between two-dimensional and three-dimensional culture systems in triple-negative breast cancer cell lines. Biochem. Biophysical Res. Commun. 533 (3), 268–274. 10.1016/j.bbrc.2020.08.075 32958246

[B102] Mulholland T. McAllister M. Patek S. Flint D. Underwood M. Sim A. (2018). Drug screening of biopsy-derived spheroids using a self-generated microfluidic concentration gradient. Sci. Rep. 8 (1), 14672. 10.1038/s41598-018-33055-0 30279484 PMC6168499

[B103] Naderi-Meshkin H. Cornelius V. A. Eleftheriadou M. Potel K. N. Setyaningsih W. A. W. Margariti A. (2023). Vascular organoids: unveiling advantages, applications, challenges, and disease modelling strategies. Stem Cell Res. Ther. 14 (1), 292. 10.1186/s13287-023-03521-2 37817281 PMC10566155

[B104] Naipal K. A. T. Verkaik N. S. Sánchez H. van Deurzen C. H. M. den Bakker M. A. Hoeijmakers J. H. J. (2016). Tumor slice culture system to assess drug response of primary breast cancer. BMC Cancer 16, 78. 10.1186/s12885-016-2119-2 26860465 PMC4748539

[B105] Nanki Y. Chiyoda T. Hirasawa A. Ookubo A. Itoh M. Ueno M. (2020). Patient-derived ovarian cancer organoids capture the genomic profiles of primary tumours applicable for drug sensitivity and resistance testing. Sci. Rep. 10 (1), 12581. 10.1038/s41598-020-69488-9 32724113 PMC7387538

[B106] Nguyen D. G. Funk J. Robbins J. B. Crogan-Grundy C. Presnell S. C. Singer T. (2016). Bioprinted 3D primary liver tissues allow assessment of organ-level response to clinical drug induced toxicity in vitro. PLoS One 11 (7), e0158674. 10.1371/journal.pone.0158674 27387377 PMC4936711

[B107] Nguyen M. De Ninno A. Mencattini A. Mermet-Meillon F. Fornabaio G. Evans S. S. (2018). Dissecting effects of anti-cancer drugs and cancer-associated fibroblasts by On-Chip reconstitution of immunocompetent tumor microenvironments. Cell Rep. 25 (13), 3884–3893.e3. 10.1016/j.celrep.2018.12.015 30590056

[B108] Noonan J. Grassia G. MacRitchie N. Garside P. Guzik T. J. Bradshaw A. C. (2019). A novel triple-cell two-dimensional model to study immune-vascular interplay in atherosclerosis. Front. Immunol. 10, 849. 10.3389/fimmu.2019.00849 31068936 PMC6491724

[B109] Nunes A. S. Barros A. S. Costa E. C. Moreira A. F. Correia I. J. (2019). 3D tumor spheroids as in vitro models to mimic *in vivo* human solid tumors resistance to therapeutic drugs. Biotechnol. Bioeng. 116 (1), 206–226. 10.1002/bit.26845 30367820

[B110] Nwokoye P. N. Abilez O. J. (2024). Bioengineering methods for vascularizing organoids. Cell Rep. Methods 4 (6), 100779. 10.1016/j.crmeth.2024.100779 38759654 PMC11228284

[B111] Ooft S. N. Weeber F. Dijkstra K. K. McLean C. M. Kaing S. van Werkhoven E. (2019). Patient-derived organoids can predict response to chemotherapy in metastatic colorectal cancer patients. Sci. Transl. Med. 11 (513), eaay2574. 10.1126/scitranslmed.aay2574 31597751

[B112] Pampaloni F. Reynaud E. G. Stelzer E. H. K. (2007). The third dimension bridges the gap between cell culture and live tissue. Nat. Rev. Mol. Cell Biol. 8 (10), 839–845. 10.1038/nrm2236 17684528

[B113] Pasch C. A. Favreau P. F. Yueh A. E. Babiarz C. P. Gillette A. A. Sharick J. T. (2019). Patient-derived cancer organoid cultures to predict sensitivity to chemotherapy and radiation. Clin. Cancer Res. 25 (17), 5376–5387. 10.1158/1078-0432.ccr-18-3590 31175091 PMC6726566

[B114] Patra B. Peng C. C. Liao W. H. Lee C. H. Tung Y. C. (2016). Drug testing and flow cytometry analysis on a large number of uniform sized tumor spheroids using a microfluidic device. Sci. Rep. 6, 21061. 10.1038/srep21061 26877244 PMC4753452

[B115] Peck R. W. Hinojosa C. D. Hamilton G. A. (2020). Organs-on-Chips in clinical pharmacology: putting the patient into the center of treatment selection and drug development. Clin. Pharmacol. Ther. 107 (1), 181–185. 10.1002/cpt.1688 31758803 PMC6977308

[B116] Pelosi A. C. Silva A. A. R. Fernandes A. Scariot P. P. M. Oliveira M. S. P. Porcari A. M. (2024). Metabolomics of 3D cell co-culture reveals alterations in energy metabolism at the cross-talk of colorectal cancer-adipocytes. Front. Med. 11, 1436866. 10.3389/fmed.2024.1436866 PMC11484090 39421865

[B117] Petersen O. W. Rønnov-Jessen L. Howlett A. R. Bissell M. J. (1992). Interaction with basement membrane serves to rapidly distinguish growth and differentiation pattern of normal and malignant human breast epithelial cells. Proc. Natl. Acad. Sci. U. S. A. 89 (19), 9064–9068. 10.1073/pnas.89.19.9064 1384042 PMC50065

[B118] Pinto M. L. Rios E. Silva A. C. Neves S. C. Caires H. R. Pinto A. T. (2017). Decellularized human colorectal cancer matrices polarize macrophages towards an anti-inflammatory phenotype promoting cancer cell invasion *via* CCL18. Biomaterials 124, 211–224. 10.1016/j.biomaterials.2017.02.004 28209528

[B119] Pittman R. N. (2011). Regulation of tissue oxygenation. San Rafael (CA): Morgan and Claypool Life Sciences. 21634070

[B120] Polidoro M. A. Ferrari E. Marzorati S. Lleo A. Rasponi M. (2021). Experimental liver models: from cell culture techniques to microfluidic organs-on-chip. Liver Int. Official J. Int. Assoc. Study Liver 41 (8), 1744–1761. 10.1111/liv.14942 33966344

[B121] Prior H. Baldrick P. De Haan L. Downes N. Jones K. Mortimer-Cassen E. (2018). Reviewing the utility of two species in general toxicology related to drug development. Int. J. Toxicol. 37 (2), 121–124. 10.1177/1091581818760564

[B122] Qu J. Kalyani F. S. Liu L. Cheng T. Chen L. (2021). Tumor organoids: synergistic applications, current challenges, and future prospects in cancer therapy. Cancer Commun. 41 (12), 1331–1353. 10.1002/cac2.12224 PMC8696219 34713636

[B123] Redmond J. McCarthy H. Buchanan P. Levingstone T. J. Dunne N. J. (2021). Advances in biofabrication techniques for collagen-based 3D *in vitro* culture models for breast cancer research. Mater. Sci. Eng. C 122, 111944. 10.1016/j.msec.2021.111944 33641930

[B124] Riffle S. Hegde R. S. (2017). Modeling tumor cell adaptations to hypoxia in multicellular tumor spheroids. J. Exp. Clin. Cancer Res. 36 (1), 102. 10.1186/s13046-017-0570-9 28774341 PMC5543535

[B125] Rimann M. Graf-Hausner U. (2012). Synthetic 3D multicellular systems for drug development. Curr. Opin. Biotechnol. 23 (5), 803–809. 10.1016/j.copbio.2012.01.011 22326911

[B126] Risangud N. Lertwimol T. Sitthisang S. Wongvitvichot W. Uppanan P. Tanodekaew S. (2024). The preparation of 3D-printed self-healing hydrogels composed of carboxymethyl chitosan and oxidized dextran via stereolithography for biomedical applications. Int. J. Biol. Macromol. 292, 139251. 10.1016/j.ijbiomac.2024.139251 39732244

[B127] Sachs N. Clevers H. (2014). Organoid cultures for the analysis of cancer phenotypes. Curr. Opin. Genet. Dev. 24, 68–73. 10.1016/j.gde.2013.11.012 24657539

[B128] Saikawa Y. Kubota T. Furukawa T. Suto A. Watanabe M. Kumai K. (1994). Single-cell suspension assay with an MTT end point is useful for evaluating the optimal adjuvant chemotherapy for advanced gastric cancer. Jpn. J. Cancer Res. Gann 85 (7), 762–765. 10.1111/j.1349-7006.1994.tb02426.x 8071118 PMC5919554

[B129] Sakuma K. Hanyu S. Takahashi H. Tanaka A. (2020). Identification of the optimal cetuximab concentration that is effective against oral squamous cell carcinoma in collagen gel droplet embedded culture drug sensitivity testing. Mol. Clin. Oncol. 12 (1), 51–56. 10.3892/mco.2019.1953 31832190 PMC6904869

[B130] Salmon S. E. Hamburger A. W. Soehnlen B. Durie B. G. Alberts D. S. Moon T. E. (1978). Quantitation of differential sensitivity of human-tumor stem cells to anticancer drugs. N. Engl. J. Med. 298 (24), 1321–1327. 10.1056/nejm197806152982401 77475

[B131] Sato T. Stange D. E. Ferrante M. Vries R. G. Van Es J. H. Van den Brink S. (2011). Long-term expansion of epithelial organoids from human Colon, adenoma, adenocarcinoma, and Barrett's epithelium. Gastroenterology 141 (5), 1762–1772. 10.1053/j.gastro.2011.07.050 21889923

[B132] Schaaf M. B. Garg A. D. Agostinis P. (2018). Defining the role of the tumor vasculature in antitumor immunity and immunotherapy. Cell Death Dis. 9 (2), 115. 10.1038/s41419-017-0061-0 29371595 PMC5833710

[B133] Schlie-Wolter S. Ngezahayo A. Chichkov B. N. (2013). The selective role of ECM components on cell adhesion, morphology, proliferation and communication in vitro. Exp. Cell Res. 319 (10), 1553–1561. 10.1016/j.yexcr.2013.03.016 23588204

[B134] Seo N. Akiyoshi K. Shiku H. (2018). Exosome-mediated regulation of tumor immunology. Cancer Sci. 109 (10), 2998–3004. 10.1111/cas.13735 29999574 PMC6172045

[B135] Shukla A. K. Yoon S. Oh S.-O. Lee D. Ahn M. Kim B. S. (2024). Advancement in cancer vasculogenesis modeling through 3D bioprinting technology. Biomimetics 9 (5), 306. 10.3390/biomimetics9050306 38786516 PMC11118135

[B136] Siolas D. Hannon GJ. (2013). Patient-derived tumor xenografts: transforming clinical samples into mouse models. Cancer Res. 73 (17), 5315–5319. 10.1158/0008-5472.can-13-1069 23733750 PMC3766500

[B137] Sivakumar R. Chan M. Shin J. S. Nishida-Aoki N. Kenerson H. L. Elemento O. (2019). Organotypic tumor slice cultures provide a versatile platform for immuno-oncology and drug discovery. Oncoimmunology 8 (12), e1670019. 10.1080/2162402x.2019.1670019 31741771 PMC6844320

[B138] Sönnichsen R. Hennig L. Blaschke V. Winter K. Körfer J. Hähnel S. (2018). Individual susceptibility analysis using patient-derived slice cultures of colorectal carcinoma. Clin. Colorectal Cancer 17 (2), e189–e199. 10.1016/j.clcc.2017.11.002 29233603

[B139] Subia B. Dahiya U. R. Mishra S. Ayache J. Casquillas G. V. Caballero D. (2021). Breast tumor-on-chip models: from disease modeling to personalized drug screening. J. Control. Release 331, 103–120. 10.1016/j.jconrel.2020.12.057 33417986 PMC8172385

[B140] Sun Q. Tan S. H. Chen Q. Ran R. Hui Y. Chen D. (2018). Microfluidic formation of coculture tumor spheroids with stromal cells as a novel 3D tumor model for drug testing. ACS Biomaterials Sci. Eng. 4 (12), 4425–4433. 10.1021/acsbiomaterials.8b00904 33418835

[B141] Szajnik M. Czystowska M. Szczepanski M. J. Mandapathil M. Whiteside T. L. (2010). Tumor-derived microvesicles induce, expand and up-regulate biological activities of human regulatory T cells (Treg). PLoS One 5 (7), e11469. 10.1371/journal.pone.0011469 20661468 PMC2908536

[B142] Takamura Y. Kobayashi H. Taguchi T. Motomura K. Inaji H. Noguchi S. (2002). Prediction of chemotherapeutic response by collagen gel droplet embedded culture-drug sensitivity test in human breast cancers. Int. J. Cancer 98 (3), 450–455. 10.1002/ijc.10208 11920599

[B143] Takasato M. Er P. X. Chiu H. S. Maier B. Baillie G. J. Ferguson C. (2015). Kidney organoids from human iPS cells contain multiple lineages and model human nephrogenesis. Nature 526 (7574), 564–568. 10.1038/nature15695 26444236

[B144] Tang M. Xie Q. Gimple R. C. Zhong Z. Tam T. Tian J. (2020b). Three-dimensional bioprinted glioblastoma microenvironments model cellular dependencies and immune interactions. Cell Res. 30 (10), 833–853. 10.1038/s41422-020-0338-1 32499560 PMC7608409

[B145] Tang Y. Xu Q. Yan M. Zhang Y. Zhu P. Li X. (2020a). Autologous culture method improves retention of tumors’ native properties. Sci. Rep. 10 (1), 20455. 10.1038/s41598-020-77238-0 33235257 PMC7686378

[B146] Tchoryk A. Taresco V. Argent R. H. Ashford M. Gellert P. R. Stolnik S. (2019). Penetration and uptake of nanoparticles in 3D tumor spheroids. Bioconjugate Chem. 30 (5), 1371–1384. 10.1021/acs.bioconjchem.9b00136 30946570

[B147] Tiriac H. Belleau P. Engle D. D. Plenker D. Deschenes A. Somerville T. D. D. (2018). Organoid profiling identifies common responders to chemotherapy in pancreatic cancer. Cancer Discov. 8 (9), 1112–1129. 10.1158/2159-8290.cd-18-0349 29853643 PMC6125219

[B148] Torisawa Y.-s. Shiku H. Kasai S. Nishizawa M. Matsue T. (2004). Proliferation assay on a silicon chip applicable for tumors extirpated from mammalians. Int. J. Cancer 109 (2), 302–308. 10.1002/ijc.11693 14750184

[B149] Torisawa Y.-s. Shiku H. Yasukawa T. Nishizawa M. Matsue T. (2005). Multi-channel 3-D cell culture device integrated on a silicon chip for anticancer drug sensitivity test. Biomaterials 26 (14), 2165–2172. 10.1016/j.biomaterials.2004.05.028 15576192

[B150] Unger C. Kramer N. Walzl A. Scherzer M. Hengstschläger M. Dolznig H. (2014). Modeling human carcinomas: physiologically relevant 3D models to improve anti-cancer drug development. Adv. Drug Deliv. Rev. 79-80, 50–67. 10.1016/j.addr.2014.10.015 25453261

[B151] Valkenburg K. C. de Groot A. E. Pienta K. J. (2018). Targeting the tumour stroma to improve cancer therapy. Nat. Rev. Clin. Oncol. 15 (6), 366–381. 10.1038/s41571-018-0007-1 29651130 PMC5960434

[B152] van de Wetering M. Francies H. E. Francis J. M. Bounova G. Iorio F. Pronk A. (2015). Prospective derivation of a living organoid biobank of colorectal cancer patients. Cell 161 (4), 933–945. 10.1016/j.cell.2015.03.053 25957691 PMC6428276

[B153] Velez D. O. Tsui B. Goshia T. Chute C. L. Han A. Carter H. (2017). 3D collagen architecture induces a conserved migratory and transcriptional response linked to vasculogenic mimicry. Nat. Commun. 8 (1), 1651. 10.1038/s41467-017-01556-7 29162797 PMC5698427

[B154] Vlachogiannis G. Hedayat S. Vatsiou A. Jamin Y. Fernandez-Mateos J. Khan K. (2018). Patient-derived organoids model treatment response of metastatic gastrointestinal cancers. Science 359 (6378), 920–926. 10.1126/science.aao2774 29472484 PMC6112415

[B155] Volkner M. Kurth T. Schor J. Ebner L. J. A. Bardtke L. Kavak C. (2021). Mouse retinal organoid growth and maintenance in longer-term culture. Front. Cell Dev. Biol. 9, 645704. 10.3389/fcell.2021.645704 33996806 PMC8114082

[B156] Walsh N. C. Kenney L. L. Jangalwe S. Aryee K.-E. Greiner D. L. Brehm M. A. (2017). Humanized mouse models of clinical disease. Annu. Rev. Pathology 12, 187–215. 10.1146/annurev-pathol-052016-100332 PMC5280554 27959627

[B157] Wang T. Green R. Howell M. Martinez T. Dutta R. Mohapatra S. (2020). The design and characterization of a gravitational microfluidic platform for drug sensitivity assay in colorectal perfused tumoroid cultures. Nanomedicine Nanotechnol. Biol. Med. 30, 102294. 10.1016/j.nano.2020.102294 32861031

[B158] Wang Y. Shi W. Kuss M. Mirza S. Qi D. Krasnoslobodtsev A. (2018). 3D bioprinting of breast cancer models for drug resistance study. ACS Biomaterials Sci. Eng. 4 (12), 4401–4411. 10.1021/acsbiomaterials.8b01277 33418833

[B159] Weeber F. van de Wetering M. Hoogstraat M. Dijkstra K. K. Krijgsman O. Kuilman T. (2015). Preserved genetic diversity in organoids cultured from biopsies of human colorectal cancer metastases. Proc. Natl. Acad. Sci. U. S. A. 112 (43), 13308–13311. 10.1073/pnas.1516689112 26460009 PMC4629330

[B160] Weigelt B. Ghajar C. M. Bissell M. J. (2014). The need for complex 3D culture models to unravel novel pathways and identify accurate biomarkers in breast cancer. Adv. Drug Deliv. Rev. 69-70, 42–51. 10.1016/j.addr.2014.01.001 24412474 PMC4186247

[B161] Weiswald L.-B. Bellet D. Dangles-Marie V. (2015). Spherical cancer models in tumor biology. Neoplasia (New York, NY) 17 (1), 1–15. 10.1016/j.neo.2014.12.004 PMC4309685 25622895

[B162] Wight T. N. Kinsella M. G. Qwarnström E. E. (1992). The role of proteoglycans in cell adhesion, migration and proliferation. Curr. Opin. Cell Biol. 4 (5), 793–801. 10.1016/0955-0674(92)90102-i 1419056

[B163] Williams E. S. Rodriguez-Bravo V. Chippada-Venkata U. De Ia Iglesia-Vicente J. Gong Y. Galsky M. (2015). Generation of prostate cancer patient derived xenograft models from circulating tumor cells. J. Vis. Exp. JoVE (105), 53182. 10.3791/53182 26555435 PMC4692658

[B164] Willoughby H. W. Maughan G. B. Tremblay P. C. Wood N. (1971). Determination of individual human tumour sensitivity to antitumour agents by tissue-slice incubation. Can. J. Surg. J. Can. De Chir. 14 (6), 406–409. 5170129

[B165] Wilson W. W. Shapiro L. P. Bradner J. M. Caudle W. M. (2014). Developmental exposure to the organochlorine insecticide endosulfan damages the nigrostriatal dopamine system in male offspring. Neurotoxicology 44, 279–287. 10.1016/j.neuro.2014.07.008 25092410 PMC4175067

[B166] Wu H. Yang Y. Bagnaninchi P. O. Jia J. (2018). Electrical impedance tomography for real-time and label-free cellular viability assays of 3D tumour spheroids. Analyst 143 (17), 4189–4198. 10.1039/c8an00729b 30070264

[B167] Xavier C. P. R. Caires H. R. Barbosa M. A. G. Bergantim R. Guimarães J. E. Vasconcelos M. H. (2020). The role of extracellular vesicles in the hallmarks of cancer and drug resistance. Cells 9 (5), 1141. 10.3390/cells9051141 32384712 PMC7290603

[B168] Xie F. Xu M. Lu J. Mao L. Wang S. (2019). The role of exosomal PD-L1 in tumor progression and immunotherapy. Mol. Cancer 18 (1), 146. 10.1186/s12943-019-1074-3 31647023 PMC6813045

[B169] Xu H. Lyu X. Yi M. Zhao W. Song Y. Wu K. (2018). Organoid technology and applications in cancer research. J. Hematol. Oncol. 11 (1), 116. 10.1186/s13045-018-0662-9 30219074 PMC6139148

[B170] Yamada K. M. Cukierman E. (2007). Modeling tissue morphogenesis and cancer in 3D. Cell 130 (4), 601–610. 10.1016/j.cell.2007.08.006 17719539

[B171] Yamaue H. Tanimura H. Kono N. Aoki Y. Tabuse K. Uchiyama K. (2003). Clinical efficacy of doxifluridine and correlation to in vitro sensitivity of anticancer drugs in patients with colorectal cancer. Anticancer Res. 23 (3B), 2559–2564. 12894541

[B172] Yan X. Zhou L. Wu Z. Wang X. Chen X. Yang F. (2019). High throughput scaffold-based 3D micro-tumor array for efficient drug screening and chemosensitivity testing. Biomaterials 198, 167–179. 10.1016/j.biomaterials.2018.05.020 29807624

[B173] Yang C. Luo J. Polunas M. Bosnjak N. Chueng S.-T. D. Chadwick M. (2020). 4D-Printed transformable tube array for high-throughput 3D cell culture and histology. Adv. Mater. 32 (40), e2004285. 10.1002/adma.202004285 32864842 PMC7603422

[B174] Yoshida G. J. (2020). Applications of patient-derived tumor xenograft models and tumor organoids. J. Hematol. Oncol. 13 (1), 4. 10.1186/s13045-019-0829-z 31910904 PMC6947974

[B175] Zhang Z. Chen Y. C. Urs S. Chen L. Simeone D. M. Yoon E. (2018). Scalable multiplexed drug-combination screening platforms using 3D microtumor model for precision medicine. Small 14 (42), e1703617. 10.1002/smll.201703617 30239130 PMC11893218

[B176] Zhao X. Xu Z. Xiao L. Shi T. Xiao H. Wang Y. (2021). Review on the vascularization of organoids and Organoids-on-a-Chip. Front. Bioeng. Biotechnol. 9, 637048. 10.3389/fbioe.2021.637048 33912545 PMC8072266

